# The apple FERONIA receptor‐like kinase MdMRLK2 negatively regulates Valsa canker resistance by suppressing defence responses and hypersensitive reaction

**DOI:** 10.1111/mpp.13218

**Published:** 2022-04-12

**Authors:** Yuanyuan Jing, Minghui Zhan, Chunrong Li, Tingting Pei, Qi Wang, Pengmin Li, Fengwang Ma, Changhai Liu

**Affiliations:** ^1^ State Key Laboratory of Crop Stress Biology for Arid Areas/Shaanxi Key Laboratory of Apple College of Horticulture Northwest A&F University Yangling Shaanxi China

**Keywords:** abscisic acid, FERONIA, hypersensitive reaction, MdMRLK2, polyphenols, salicylic acid, *Valsa* canker

## Abstract

Valsa canker, caused by the fungus *Valsa mali*, is one of the most destructive diseases of apple trees in China and other East Asian countries. The plant receptor‐like kinase FERONIA is involved in plant cell growth, development, and immunity. However, little is known about the function of FERONIA in apple defence against *V*. *mali*. In this study, we found that *MdMRLK2* was highly induced by *V. mali* in twigs of *V. mali‐*susceptible *Malus mellana* but not in those of the resistant species *Malus yunnaensis*. 35S:*MdMRLK2* apple plants showed compromised resistance relative to wild‐type (WT) plants. Further analyses indicated that 35S:*MdMRLK2* apple plants had enhanced abscisic acid (ABA) levels and reduced salicylic acid (SA) levels relative to the WT on *V. mali* infection. *MdMRLK2* overexpression also suppressed polyphenol accumulation and inhibited the activities of phenylalanine ammonia‐lyase (PAL), β‐1,3‐glucanase (GLU), and chitinase (CHT) during *V. mali* infection. Moreover, MdMRLK2 interacted with MdHIR1, a hypersensitive‐induced response protein, and suppressed the MdHIR1‐mediated hypersensitive reaction (HR), probably by impairing MdHIR1 self‐interaction. Collectively, these findings demonstrate that overexpression of *MdMRLK2* compromises Valsa canker resistance, probably by (a) altering ABA and SA levels, (b) suppressing polyphenol accumulation, (c) inhibiting PAL, GLU, and CHT activities, and (d) blocking MdHIR1‐mediated HR by disrupting MdHIR1 self‐interaction.

## Graphical abstract

An apple FERONIA receptor‐like kinase, MdMRLK2, negatively regulates Valsa canker resistance by altering abscisic acid and salicylic acid levels, suppressing polyphenol accumulation, defence enzymes activities, and the hypersensitive reaction.

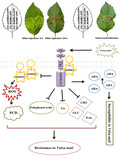

## 1 INTRODUCTION

Apple (*Malus domestica*) is a popular temperate fruit that has long been appreciated for its unique characteristics and rich nutrition (Daccache et al., 2020; Sun et al., 2020). China is the largest producer of apples worldwide (Wang et al., 2018). Apple trees are highly vulnerable to many diseases, including those caused by fungi, such as Marssonina blotch (*Diplocarpon mali*) (Zhao et al., 2013), powdery mildew (*Podosphaera leucotricha*) (Tian et al., 2019), bitter rot (*Colletotrichum acutatum*) (Jurick et al., 2011), and Valsa canker (*Valsa mali*) (Wang et al., 2011a, 2011b). Valsa canker caused by *V. mali* occurs with an annual average incidence of approximately 52.7% (Meng et al., 2019). Valsa canker is widespread and destructive: it causes the death of twigs, limbs, and finally the whole tree, reducing apple production and causing significant economic losses in China (Song et al., 2020). However, the molecular defence mechanisms of apple plants against *V. mali* infection are poorly understood, and only a few effective management strategies have been reported to date.

Plant resistance to pathogens is a dynamic and complex biological process that involves various changes at the biochemical, molecular, and physiological levels (AbuQamar et al., 2017). When a plant detects an attempted pathogen invasion, it rapidly activates sophisticated defence mechanisms to protect itself from foreign threats. The activation of complex phytohormone signalling networks is vital, as it stimulates the plant immune signalling network (Robert‐Seilaniantz et al., 2011; Yang et al., 2015). Salicylic acid (SA) is an important plant hormone that plays a critical role in plant disease resistance either by promoting the synthesis of preformed or inducible antimicrobial defence compounds termed phytoalexins or by activating defence signalling (Kumar, 2014; Vlot et al., 2009). SA is required for the activation of systemic acquired resistance, which is marked by increased expression of many defence proteins, including pathogenesis‐related (PR) proteins (Kumar, 2014). Systemic acquired resistance is a form of systemic immunity that protects distal, uninfected parts of the plant against secondary infections by related or unrelated pathogens (Kachroo et al., 2020). Plants deficient in SA signalling are incapable of developing systemic acquired resistance and do not show pathogenesis‐related gene activation on pathogen infection (Pieterse et al., 2009). Li et al. (2019a) demonstrated that exogenous SA improved tomato resistance to tomato yellow leaf curl virus. Application of exogenous SA has also been reported to induce resistance to Glomerella leaf spot in apple cv. Gala leaves (Zhang et al., 2016). In addition to SA, other plant hormones such as jasmonic acid (JA) and abscisic acid (ABA) also trigger and modulate plant resistance to biotrophic and necrotrophic pathogens through a complex signalling network. JA plays an essential role in plant defence responses against pathogens, especially fungi (Zhang et al., 2020). Mutants of JA biosynthesis and signalling genes display increased susceptibility to various fungi, and studies have shown that SA acts antagonistically to JA (Mur et al., 2006). Whether or not the JA signalling pathway is enhanced also depends on the lifestyle of the pathogen. ABA has been shown to enhance susceptibility in other plant–pathogen systems (Adie et al., 2007). For example, early studies showed that pretreatment of potato plants with ABA increased their susceptibility to *Phytophthora infestans* and *Cladosporium cucumerinum* (Henfling et al., 1980). In wheat, *Puccinia striiformis* f. sp. *tritici* stimulates ABA accumulation, also promoting fungal infection (Huai et al., 2019).

Phenolic compounds are important plant secondary metabolites whose production by the shikimate‐phenylpropanoid pathways is enhanced under stress conditions (Rasouli et al., 2016). Phenolic compounds are important for the induction of plant resistance (Mandal et al., 2010). For example, the synthesis of phenolic compounds is triggered in cells adjacent to injured tissues to restrict pathogen spread from local sites (Ferreira et al., 2007). In particular, phenolic acids, the main components of phenolic compounds, are ubiquitous in plants and can be incorporated into the cell wall in response to biotic stress (Oliveira et al., 2020; Zafari et al., 2016). Some plants respond to pathogen attack by accumulating phenolic acids such as gallic, ferulic, *p*‐coumaric, and chlorogenic acids. Studies have shown that some phenolic acids are frequently involved in the plant defence system; one example is *p*‐coumaric acid, which was reported to be positively correlated with fungal incidence (Giorni et al., 2020). In addition to phenolic acids, defence‐related enzymes including phenylalanine ammonia‐lyase (PAL), β‐1,3‐glucanase (GLU), and chitinase (CHT) are also induced to defend against *Fusarium sulphureum* in potato (Yu et al., 2016). Additional studies have shown that PAL, GLU, and CHT activities increase significantly in the presence of *Pseudoperonospora cubensis* and enhance the resistance of cucumber leaves (Shi et al., 2007). Tian et al. (2006) reported that elicitors significantly enhanced defence‐related enzyme activities to defend against Alternaria rot in pear.

Phytopathogenic microorganisms are common in nature and pose a constant threat to plants. Nonetheless, plants rarely become infected and develop disease; a multilayered innate immune system protects them from most pathogens (Johansson et al., 2015). To cope with pathogens, infected plants may deploy a rapid and strong defensive response called the hypersensitive reaction (HR) (Balint‐Kurti, 2019). The HR is a local cell death response at the site of infection that involves highly dynamic reorganization of host cells and often manifests as localized programmed cell death (PCD), which effectively prevents the spread of biotrophic pathogens (Balint‐Kurti, 2019; Higaki et al., 2011; Liu et al., 2005). An HR typically occurs during successful defence in host plants, usually leaving only small necrotic spots (Wang et al., 2016). Wang et al. (2009) observed that *Arabidopsis thaliana* with delayed HR showed compromised resistance to *Pseudomonas syringae* pv. *tomato* (Pto) DC3000. Studies also showed that HR inhibition allowed *Phaerotheca fuliginea* to penetrate and form haustoria in wheat (Li et al., 2010). Although significant research efforts have focused on the regulation of plant HR, many questions about potential mechanisms remain to be addressed. Plants employ multiple mechanisms to suppress the inappropriate activation of HR and to constrain it after activation because of its potentially severe costs (Balint‐Kurti, 2019). Previous studies have demonstrated that HIR1 associates with the plasma membrane and triggers hypersensitive cell death in rice and pepper (Choi et al., 2011; Zhou et al., 2010). Plants have evolved an intricate system to control HIR1‐mediated HR, and among the negative regulators of this response are the so‐called leucine‐rich repeat (LRR) proteins (Jung & Hwang, 2007). Small LRR proteins have been reported to negatively modulate HIR1‐mediated HR during pathogen attack (Choi et al., 2011). LRR domains exist in most receptor‐like kinases (RLKs) and participate in signal transduction for disease resistance (Hosseini et al., 2020). Plant RLKs and receptor‐like proteins can rapidly recognize invading pathogens (Zhao et al., 2019). Recently, many researchers have reported that the RLK FERONIA is involved in plant responses to pathogen invasion (Liao et al., 2017). For example, *fer* mutants have been shown to be less susceptible to the powdery mildew *Golovinomyces orontii* (Kessler et al., 2010). Pathogenic fungi produce RALF (Rapid Alkalinization Factor) 1‐like peptide to activate FER signalling events, including apoplastic alkalinization, that in turn activate Fmk1 in the fungus to enhance virulence (Masachis et al., 2016).

FERONIA, a receptor for the RALF peptide ligand, integrates a number of regulatory pathways that target cell expansion, energy metabolism, immune responses, and abiotic stress responses (Liao et al., 2017; Stegmann et al., 2017). Previous studies have shown that some pathogenic fungi produce RALF‐like peptides to activate the host FERONIA‐mediated pathway and thus increase their virulence and cause plant disease (Liao et al., 2017). Although many studies suggest that FERONIA is involved in immune responses in a complex with other proteins (Masachis et al., 2016; Xiao et al., 2019), no direct evidence has yet been provided on the role of FERONIA in apple defence against *V. mali* infection. By evaluating the expression profile of an apple FERONIA receptor‐like kinase gene *MdMRLK2* in response to *V. mali*, we discovered that *V. mali* infection rapidly triggered the strong up‐regulation of *MRLK2* in a *V. mali‐*susceptible species *Malus mellana*, but not in a resistant species *Malus yunnaensis*, suggesting that *MRLK2* may negatively regulate *V. mali* resistance in apple. Therefore, in this study, we characterized the function and mechanism of *MdMRLK2* in apple defence responses against *V. mali* infection and show that *MdMRLK2* overexpression alters phytohormone levels, suppresses polyphenol accumulation, and inhibits the activities of defence‐related enzymes. Our results also demonstrate that MdMRLK2 interacts with MdHIR1 (NCBI no. LOC103428302) and limits MdHIR1‐mediated HR, which by impairing MdHIR1 self‐interaction ultimately compromises Valsa canker resistance.

## 2 RESULTS

### 2.1 Overexpression of *MdMRLK2* negatively regulated apple *V. mali* resistance

**FIGURE 1:**
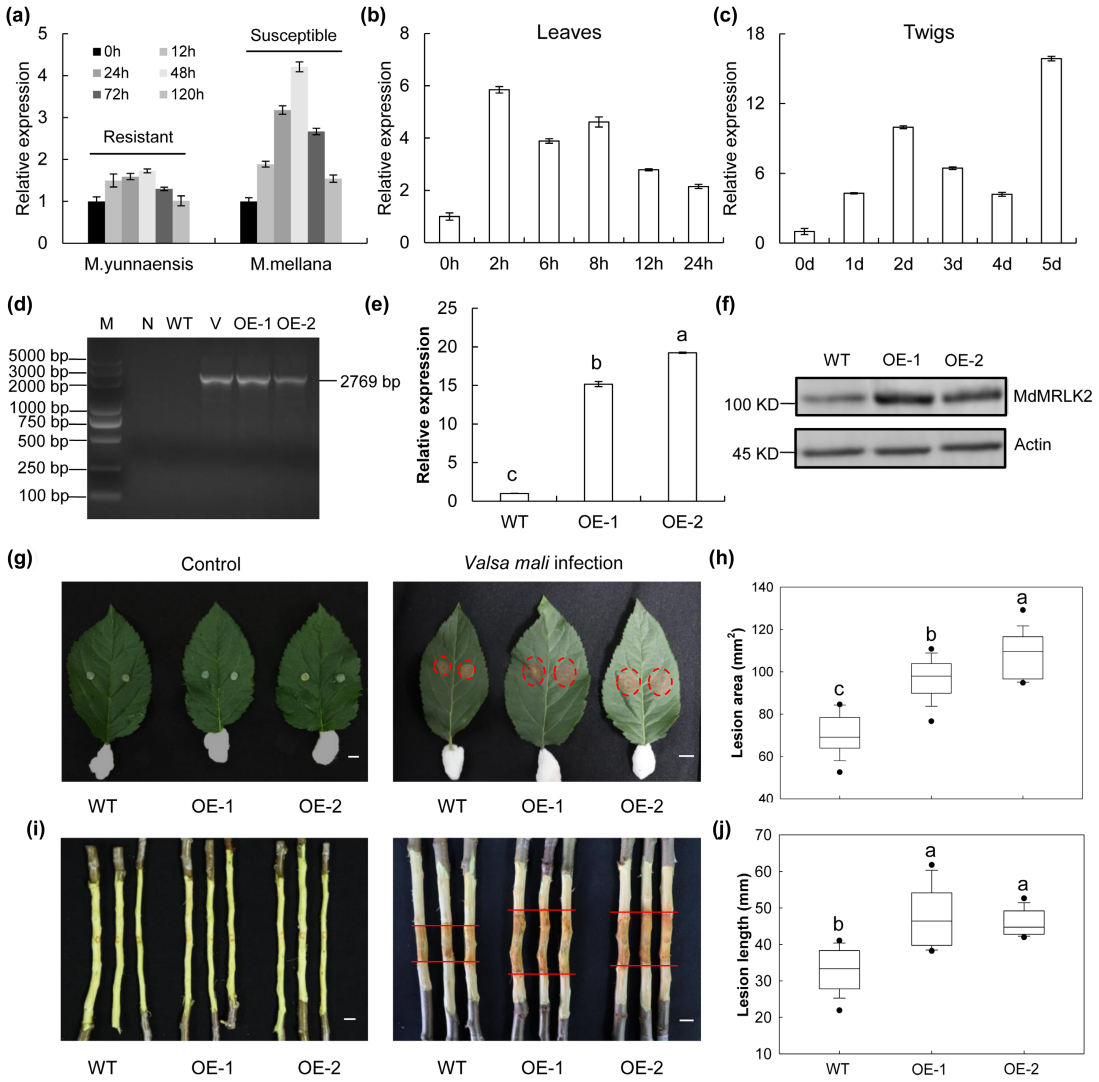
Overexpression of *MdMRLK2* negatively regulated apple resistance to *Valsa mali*. (a) Expression profile of *MRLK2* in twigs of *V. mali‐*resistant and ‐susceptible *Malus* species after inoculation with *V. mali*. Expression of *MdMRLK2* in leaves (b) and twigs (c) of the susceptible apple cultivar Gala‐3 after inoculation with *V. mali*. (d) PCR confirmation of transgenic apple plants. Lanes: M, molecular marker DL5000; N, negative control; WT, nontransformed wild type; V, vector containing pBI121‐MdMRLK2; OE‐1 and OE‐2, 35S:*MdMRLK2*‐transgenic apple lines. (e) Reverse transcription‐quantitative PCR analysis of *MdMRLK2* transcripts in the leaves of lines OE‐1 and OE‐2. (f) The protein level of MdMRLK2 in WT, OE‐1, and OE‐2 apple plants. (g) Phenotypes of leaves from WT and 35S:*MdMRLK2* plants inoculated with *V. mali* at 3 days postinoculation (dpi). (h) Lesion areas in leaves of WT and OE‐1 and OE‐2 plants. (i) Phenotypes of twigs from WT and OE‐1 and OE‐2 plants inoculated with *V. mali* at 5 dpi. (j) Lesion lengths on twigs of WT and OE‐1 and OE‐2 plants. Data are the means ± *SE* of 15 biological replicates. Different letters indicate significant differences between treatments based on one‐way analysis of variance and Tukey's multiple comparison test (*p* < 0.05)

The expression of *MRLK2* was highly induced on *V. mali* infection in twigs of *M. mellana* but not of *M. yunnaensis* (Figure 1a), suggesting that it may play a negative role in resistance. We examined the expression profile of *MdMRLK2* in the *V. mali‐*susceptible cultivar Gala‐3 (GL‐3) and found that *MdMRLK2* was rapidly and strongly induced in leaves and twigs on *V. mali* infection (Figure 1b,c). To explore the function of *MdMRLK2* in apple defence against *V. mali*, we generated two 35S:*MdMRLK2* transgenic lines, OE‐1 and OE‐2, with *MdMRLK2* expression levels that were increased 15.2‐ and 19‐fold, respectively (Figure 1d,e). The protein level of MdMRLK2 in wild‐type (WT), OE‐1, and OE‐2 apple plants was analysed, which clearly showed that the two OE lines expressed full‐length MdMRLK2 and the MdMRLK2 bands were stronger in OE lines than in WT plants (Figure 1f). In addition, we inoculated leaves and twigs of WT and OE lines with *V. mali*. Three days after inoculation, the lesion areas were clearly larger in the OE lines than in WT plants (Figure 1g,h). By 5 days postinoculation (dpi), the twig lesion lengths were significantly longer in OE lines than in WT plants (Figure 1i,j). We also inoculated three *MdMRLK2* RNAi apple calli lines with *V. mali*. The lesion areas were significantly larger in WT calli than in the *MdMRLK2* RNAi lines, and the WT calli showed more cell death than the *MdMRLK2* RNAi lines based on trypan blue staining (Figure S1). These results indicated that MdMRLK2 plays a negative role in *V. mali* resistance.

### 2.2 Overexpression of *MdMRLK2* increased ABA but reduced SA content of apple plants on *V. mali* infection

**FIGURE 2:**
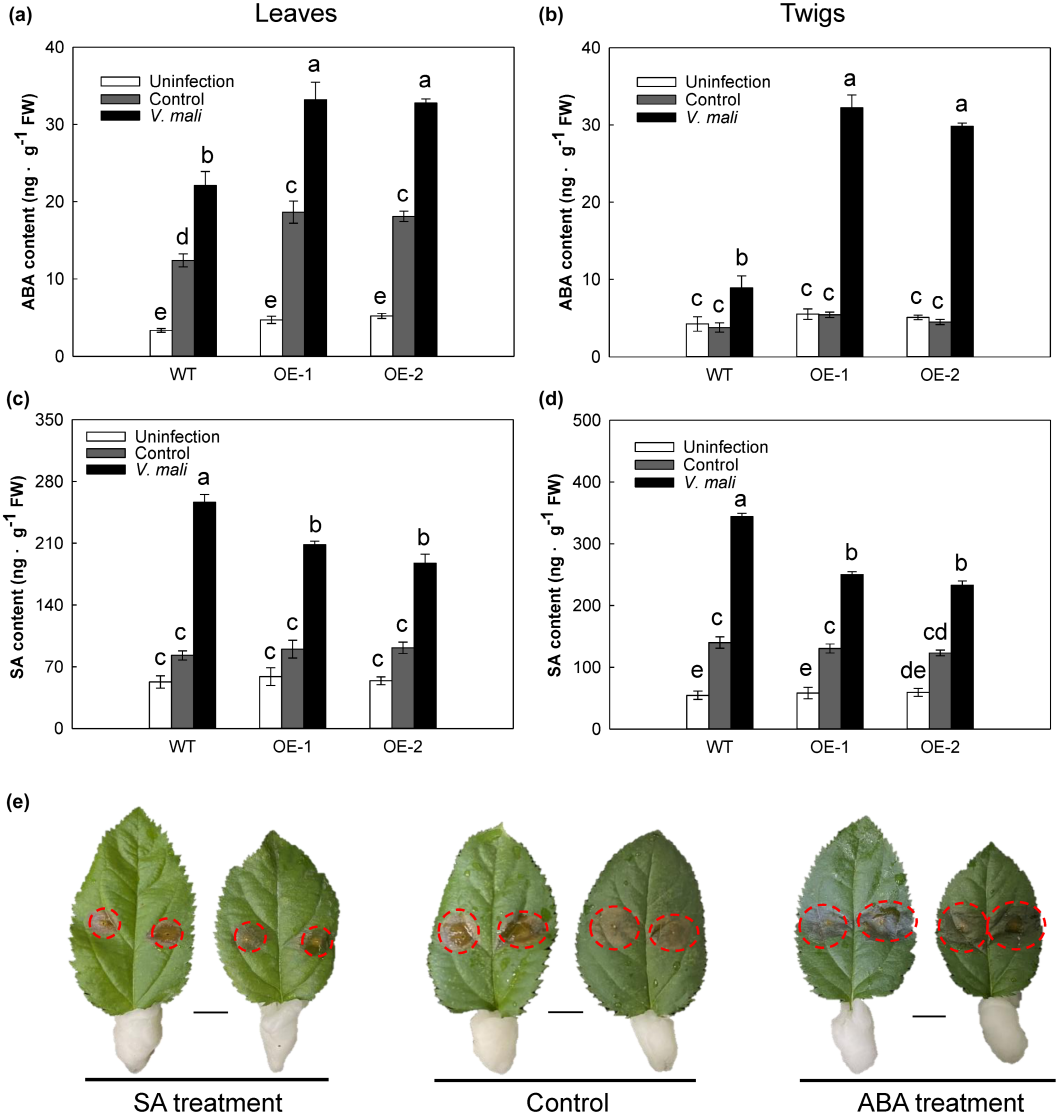
The hormones level of wild‐type (WT) and 35S:*MdMRLK2* (OE‐1 and OE‐2) apple plants after inoculation with *Valsa mali*. Abscisic acid (ABA) levels in (a) leaves and (b) twigs of WT and 35S:*MdMRLK2* plants. Salicylic acid (SA) levels in (c) leaves and (d) twigs of WT and 35S:*MdMRLK2* plants. (e) WT leaves inoculated with *V. mali* after spraying with 100 μM ABA or 300 μM SA for 6 h. Data are the means ± *SE* of three biological replicates. Different letters indicate significant differences between treatments based on one‐way analysis of variance and Tukey's multiple comparison test (*p* < 0.05)

We next measured the contents of three hormones with important roles in disease resistance: ABA, SA, and JA. There were no differences in ABA content between WT and OE lines at day 0, but at 3 dpi the ABA level was 50.2% higher in leaves of 35S:*MdMRLK2* lines than in leaves of WT plants (Figure 2a). In twigs, the level of ABA was 3.6‐fold and 3.3‐fold higher in OE‐1 and OE‐2 plants, respectively, compared with the WT (Figure 2b). By contrast, the leaf SA content was 18.8% and 26.9% lower in OE‐1 and OE‐2 than in WT plants (Figure 2c), and the SA content in twigs showed a similar trend (Figure 2d). Plant resistance‐related genes such as *PR1*, *PR4*, *PR5*, and *PAL* were expressed at higher levels in leaves and twigs of WT plants than in those of OE lines (Figure S2). There was no significant difference in JA content between 35S:*MdMRLK2* lines and WT plants (Figure S3). To verify the effects of ABA and SA in *V. mali* resistance, ABA and SA were sprayed on leaves before inoculation with *V. mali*; the lesion areas were clearly larger following ABA treatment and smaller following SA treatment compared with those of the controls (Figure 2e).

### 2.3 Overexpression of *MdMRLK2* reduced the polyphenol content of apple plants in response to *V. mali* infection

**FIGURE 3:**
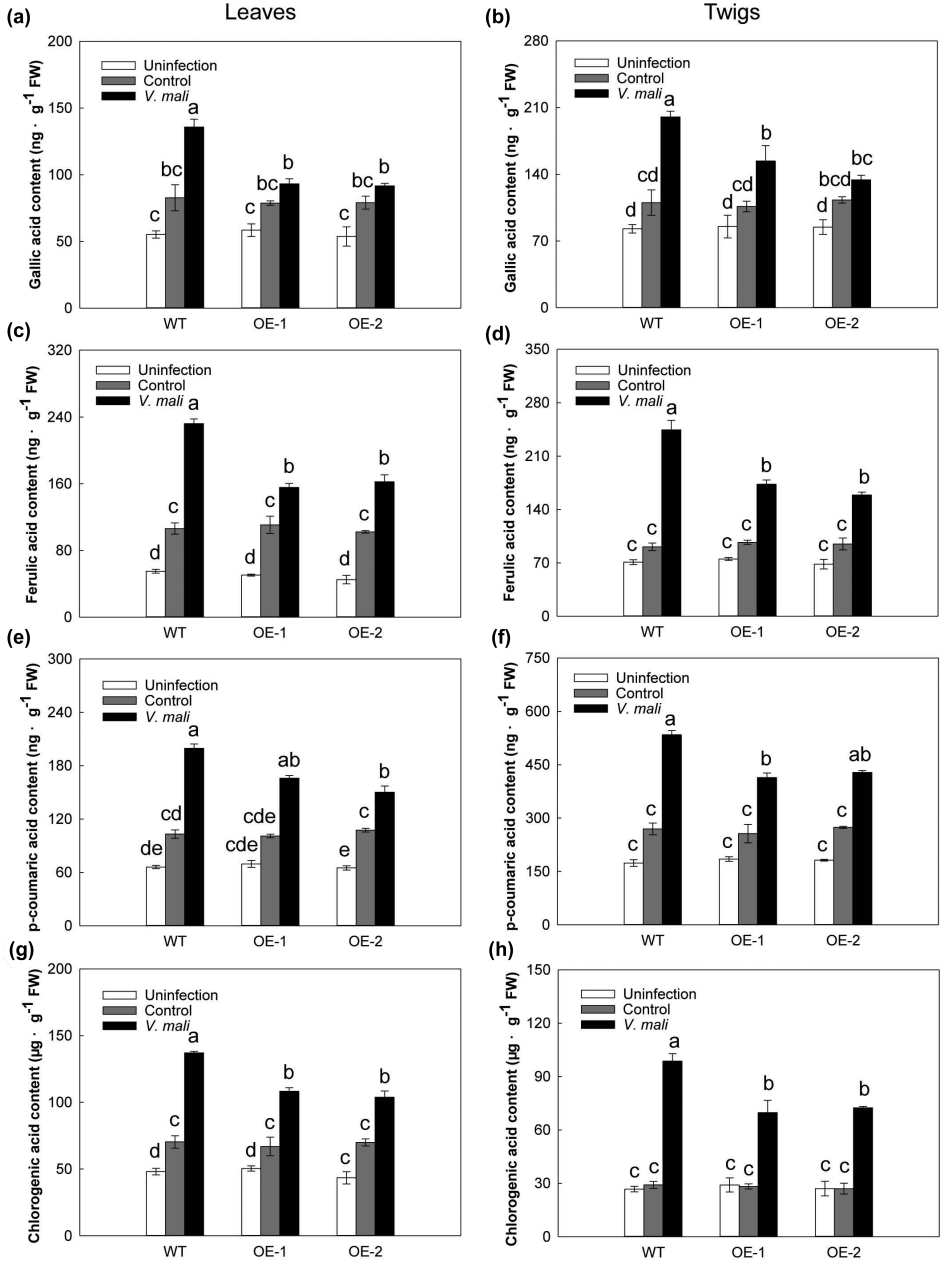
Polyphenol levels of wild‐type (WT) and 35S:*MdMRLK2* (OE‐1 and OE‐2) apple plants after inoculation with *Valsa mali*. Gallic acid contents in (a) leaves and (b) twigs of WT and 35S:*MdMRLK2* plants. Ferulic acid contents in (c) leaves and (d) twigs of WT and 35S:*MdMRLK2* plants. *p*‐Coumaric acid contents in (e) leaves and (f) twigs of WT and 35S:*MdMRLK2* plants. Chlorogenic acid contents in (g) leaves and (h) twigs of WT and 35S:*MdMRLK2* plants. Data are the means ± *SE* of three biological replicates. Different letters indicate significant differences between treatments based on one‐way analysis of variance and Tukey's multiple comparison test (*p* < 0.05)

Polyphenol concentration in plant tissues is a good predictor of plant stress tolerance (Abedi et al., 2020). Hence, we determined the contents of gallic acid, ferulic acid, *p*‐coumaric acid, and chlorogenic acid in leaves and twigs of the WT and OE lines. Polyphenol content increased in both leaves and twigs after inoculation with *V. mali*, but the increase in polyphenol content was significantly higher in the WT than in the transgenic lines (Figure 3a–h). This result suggests that *MdMRLK2* plays a negative role in regulating polyphenol accumulation in response to *V. mali* infection.

### 2.4 Overexpression of *MdMRLK2* inhibited PAL, GLU, and CHT activities during *V. mali* infection in apple plants

**FIGURE 4:**
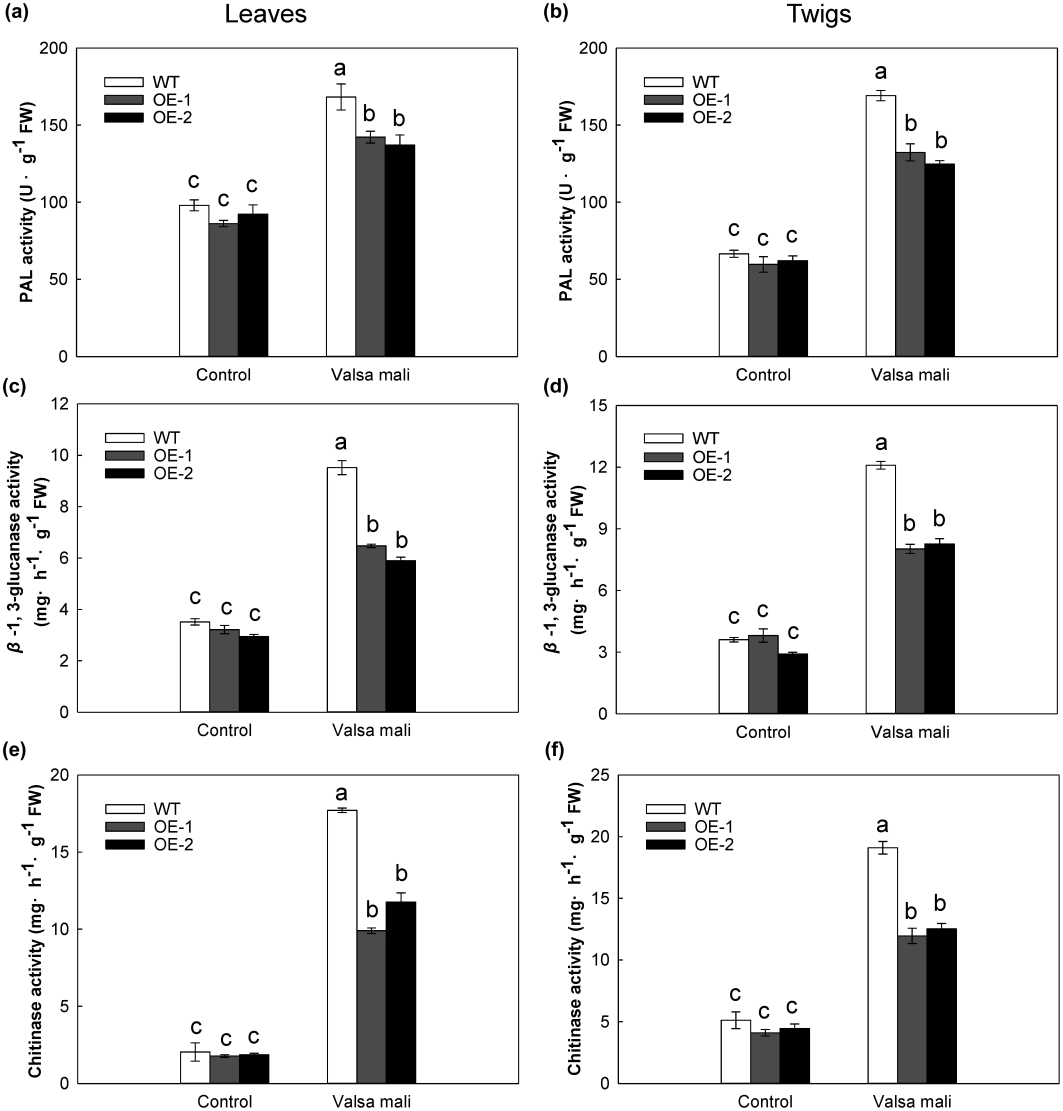
Activities of phenylalanine ammonia‐lyase (PAL), β‐1,3‐glucanase (GLU), and chitinase (CHT) in wild‐type (WT) and 35S:*MdMRLK2* (OE‐1 and OE‐2) plants after inoculation with *Valsa mali*. PAL activity of (a) leaves and (b) twigs in WT and 35S:*MdMRLK2* plants. GLU activity of (c) leaves and (d) twigs in WT and 35S:*MdMRLK2* plants. CHT activity of (e) leaves and (f) twigs in WT and 35S:*MdMRLK2* plants. Data are the means ± *SE* of three biological replicates. Different letters indicate significant differences between treatments based on one‐way analysis of variance and Tukey's multiple comparison test (*p* < 0.05)

Further analyses were performed to determine whether the activities of disease‐related enzymes increased after *V. mali* inoculation. Leaf PAL activity was significantly increased by 71.7% in WT plants but by only 62.2% in OE‐1 and 48.6% in OE‐2 (Figure 4a). Similarly, the PAL activity in WT twigs was 27.8% and 35.7% higher than that in OE‐1 and OE‐2 twigs, respectively (Figure 4b). Leaf GLU activity was increased 2.71‐fold in WT plants but only 2.0‐fold in OE‐1 and OE‐2 plants (Figure 4c), and twig GLU activity in WT plants was 1.5 times higher than that in OE lines (Figure 4d). CHT activity in both leaves and twigs increased to a greater extent in WT plants than in OE lines after *V. mali* infection (Figure 4e,f).

### 2.5 MdMRLK2 interacted with MdHIR1 and limited the HR mediated by MdHIR1

**FIGURE 5:**
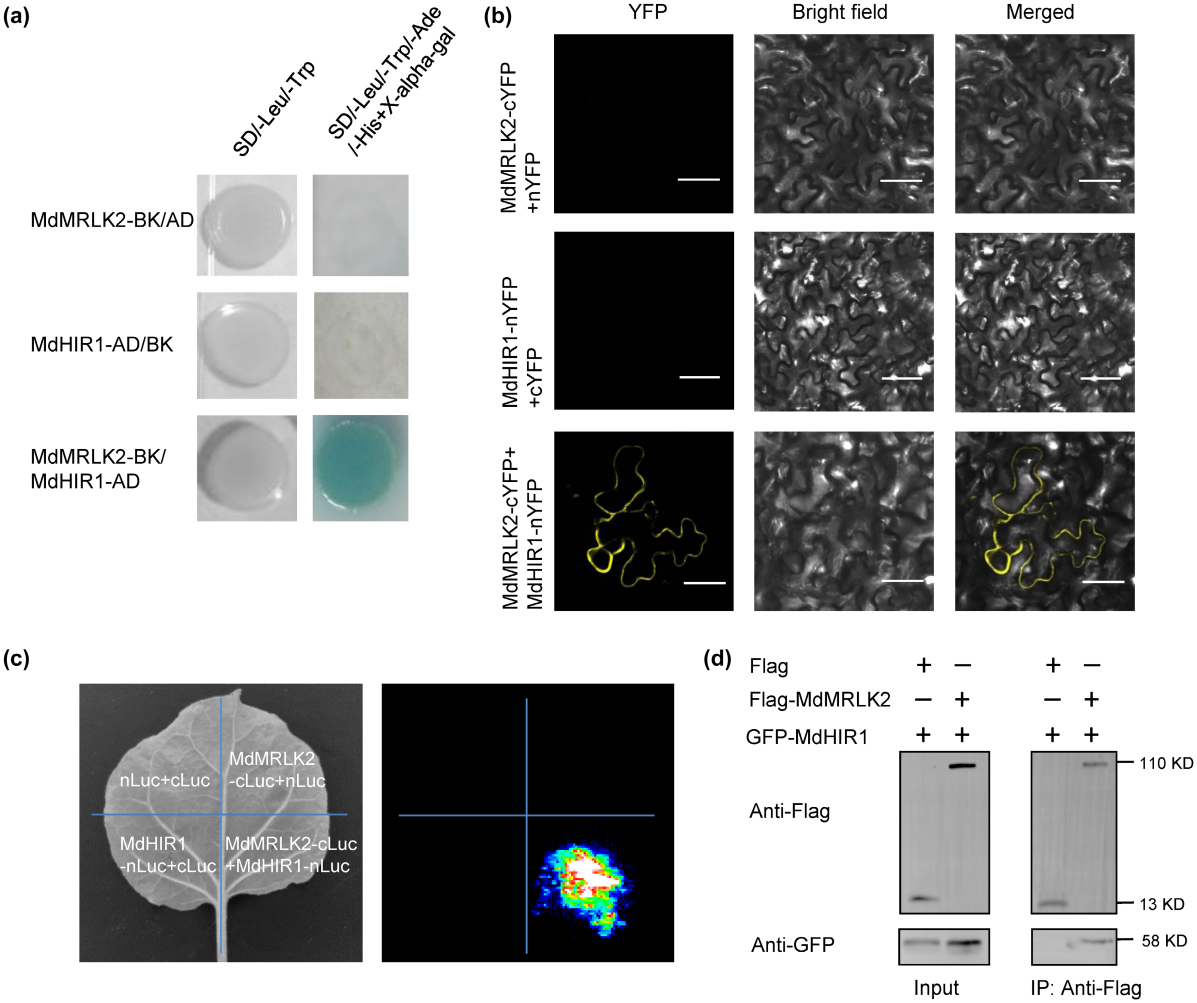
The interaction between MdMRLK2 and MdHIR1. (a) A yeast two‐hybrid assay showed the interaction between MdMRLK2 and MdHIR1. (b) Confirmation of the interaction between MdMRLK2 and MdHIR1 by bimolecular fluorescence complementation assay in *Nicotiana benthamiana* epidermal cells as indicated by a yellow fluorescence signal. nYFP, the construct for YFP N‐terminal fusion expression; cYFP, the construct for YFP C‐terminal fusion expression. Bar = 50 μm. (c) Confirmation of the interaction between MdMRLK2 and MdHIR1 by a split‐luciferase complementation assay; the combinations of cLUC with nLUC, MdMRLK2‐cLUC with nLUC, and MdHIR1‐nLUC with cLUC served as negative controls. (d) A co‐immunoprecipitation assay showed the interaction between MdMRLK2 and MdHIR1

**FIGURE 6:**
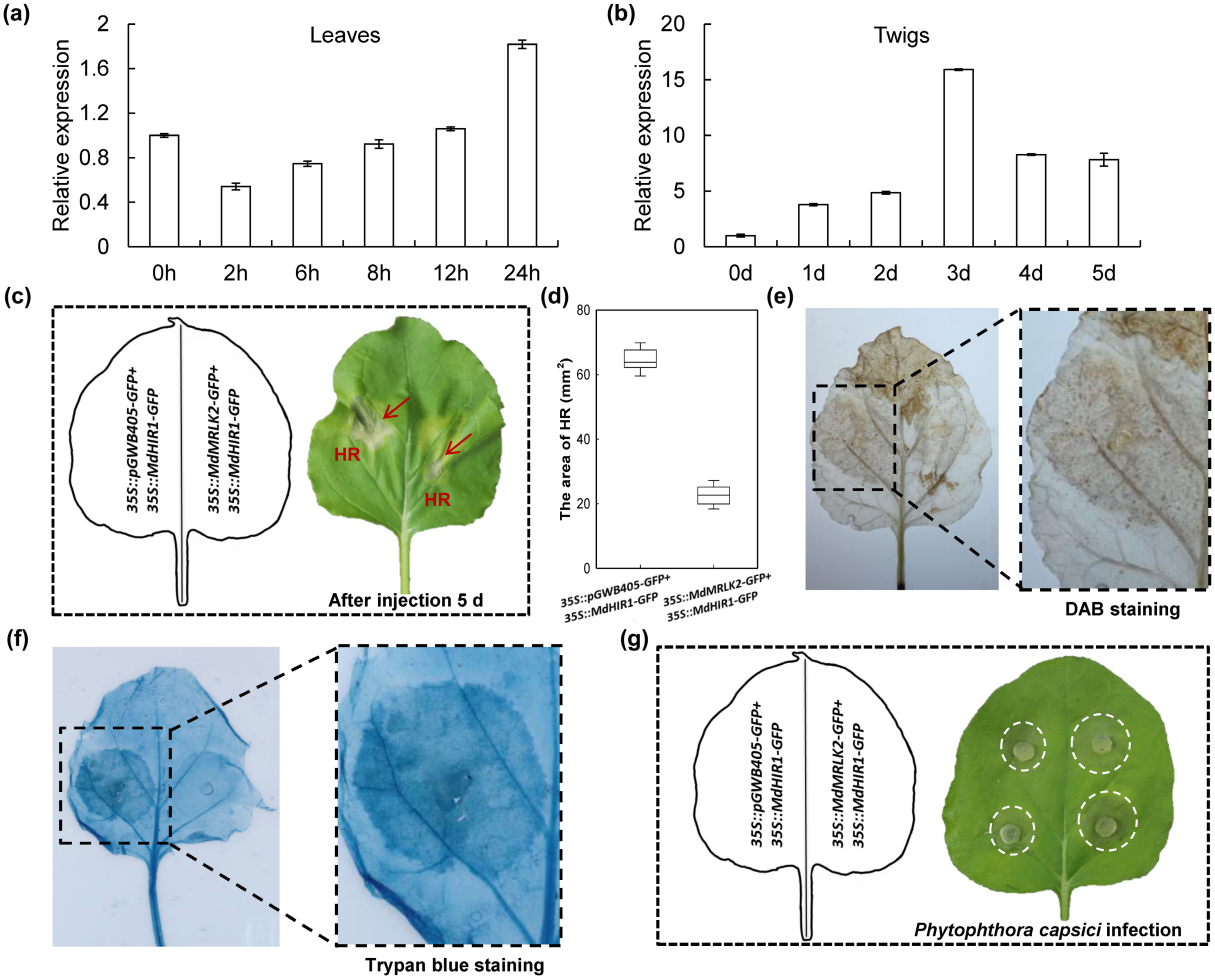
Overexpression of *MdMRLK2* limited the MdHIR1‐mediated hypersensitive reaction (HR) in *Nicotiana benthamiana* leaves. Expression of *MdHIR1* in apple (a) leaves and (b) twigs after inoculation with *Valsa mali*. (c) Co‐expression of 35S:*MdMRLK2‐GFP* with 35S:*MdHIR1‐GFP* suppressed HR 5 days after agroinfiltration; 20 plants were used as biological replicates for this experiment. (d) The area of HR in *N. benthamiana* leaves 5 days after agroinfiltration. (e) H_2_O_2_ accumulation assessed by staining with 3,3′‐diaminobenzidine‐HCl (DAB) after agroinfiltration for 48 h; 10 leaves from five 1‐month‐old *N. benthamiana* plants were used as biological replicates. (f) Cell death stained with trypan blue after agroinfiltration for 48 h; 10 leaves from five 1‐month‐old *N. benthamiana* plants were used as biological replicates. (g) Co‐expression of 35S:*MdMRLK2‐GFP* with 35S:*MdHIR1‐GFP* reduced *Phytophthora capsici* resistance in *N. benthamiana*, 20 leaves from five 1‐month‐old *N. benthamiana* plants were used as biological replicates

To explore the molecular mechanism by which MdMRLK2 compromised *V. mali* resistance, we performed yeast two‐hybrid (Y2H) screening and found that MdMRLK2 targeted hypersensitive‐induced response protein‐like protein 1 (MdHIR1) (Figure 5a). A bimolecular fluorescence complementation (BiFC) assay confirmed their interaction (Figure 5b). No fluorescence signal in the yellow fluorescent protein (YFP) channel was observed when MdMRLK2‐cYFP and a plasma membrane‐localized aquaporin MdPIP2‐nYFP were co‐expressed (Figure S4). We also performed a split‐luciferase complementation assay in *Nicotiana* *benthamiana* leaves; the co‐expression of MdMRLK2‐cLUC and MdHIR1‐nLUC reconstituted luciferase activity, and the expression of cLUC with nLUC, MdMRLK2‐nLUC with cLUC, and MdHIR1‐nLUC with cLUC served as negative controls (Figure 5c). A co‐immunoprecipitation assay also indicated that MdMRLK2 interacted with MdHIR1 (Figure 5d). A subcellular localization assay indicated that MdHIR1 was localized to the plasma membrane (Figure S5). In addition, the relative expression of *MdHIR1* in leaves and twigs was shown to be responsive to *V. mali* infection (Figure 6a,b).

**FIGURE 7:**
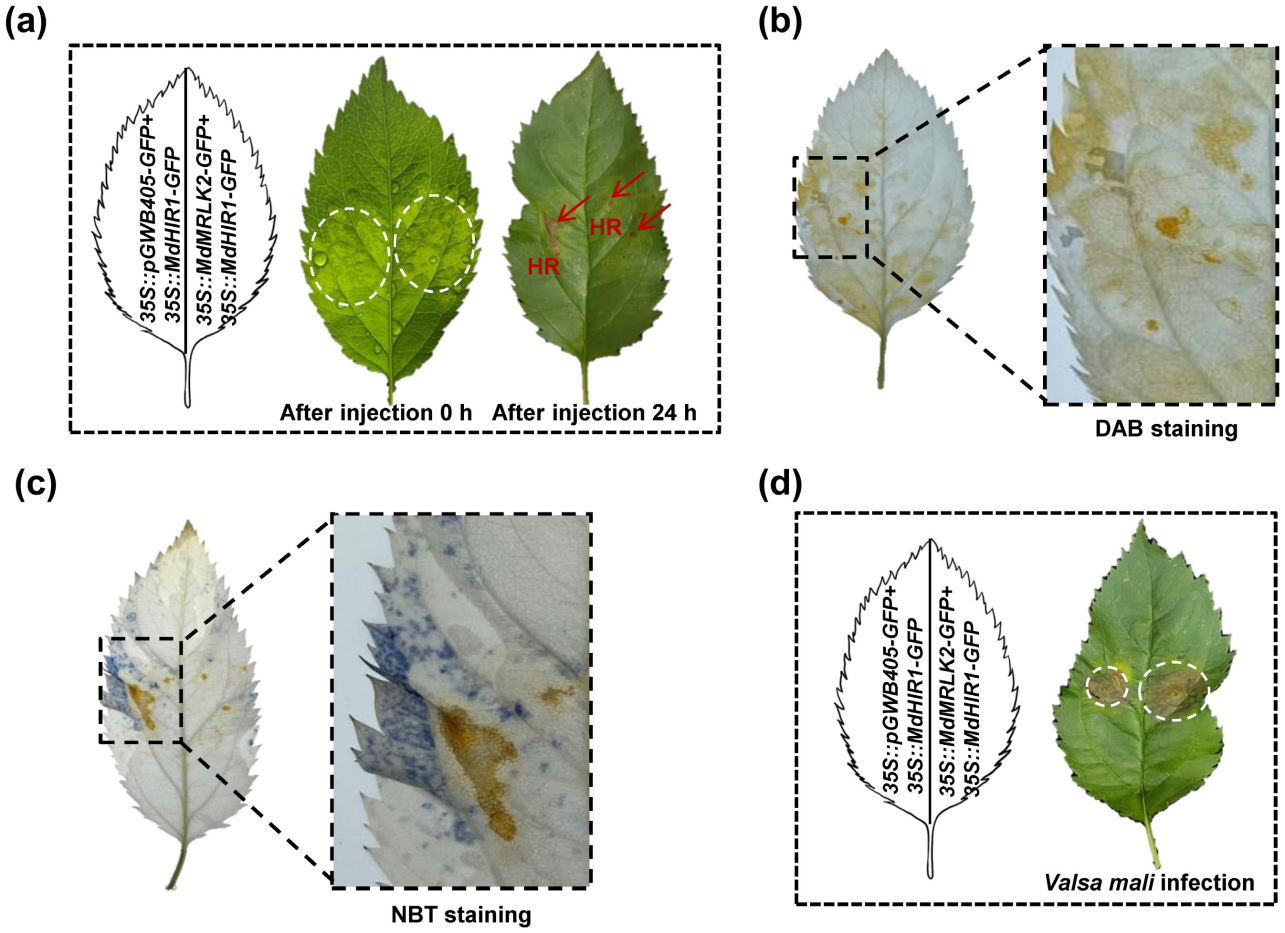
Overexpression of *MdMRLK2* limited the MdHIR1‐mediated hypersensitive reaction (HR) in apple leaves. (a) Co‐expression of 35S:*MdMRLK2‐GFP* with 35S:*MdHIR1‐GFP* suppressed HR 24 h after agroinfiltration in apple leaves. (b) H_2_O_2_ accumulation assay by 3,3′‐diaminobenzidine‐HCl (DAB) staining after agroinfiltration for 24 h; 10 leaves from five 2‐month‐old apple plants were used as biological replicates. (c) ∙O_2_^−^ accumulation assessed by staining with nitrotetrazolium blue chloride (NBT) after agroinfiltration for 24 h; 10 leaves from five 2‐month‐old apple plants were used as biological replicates. (d) Co‐expression of 35S:*MdMRLK2‐GFP* with 35S:*MdHIR1‐GFP* reduced *Valsa mali* resistance in apple; 20 leaves from ten 2‐month‐old apple plants were used as biological replicates

Why was the interaction of MdMRLK2 with MdHIR1 not accompanied by *V. mali* resistance? We hypothesized that the binding of MdMRLK2 to MdHIR1 might interfere with the MdHIR1‐mediated HR. To test this hypothesis, we co‐expressed 35S:*GFP* (green fluorescent protein) with 35S:*MdHIR1‐GFP* and 35S:*MdMRLK2‐GFP* with 35S:*MdHIR1‐GFP* in *N. benthamiana* and apple leaves. Interestingly, co‐expression of 35S:*MdMRLK2‐GFP* with 35S:*MdHIR1‐GFP* limited the MdHIR1‐mediated hypersensitive reaction, and the HR area was clearly smaller in leaves that co‐expressed 35S:*MdMRLK2‐GFP* with 35S:*MdHIR1‐GFP* (right side of leaf) than in leaves that co‐expressed 35S:*GFP* with 35S:*MdHIR1‐GFP* (left side of leaf) in both *N*. *benthamiana* and apple (Figures 6c,d and 7a). We then measured the accumulation of reactive oxygen species (ROS) in *MdHIR1*‐overexpressing *N. benthamiana* and apple leaves. As we expected, the *MdHIR1*‐induced ROS accumulation was much lower in leaves that co‐expressed 35S:*MdMRLK2‐GFP* with 35S:*MdHIR1‐GFP* (right side of leaf) than in those that co‐expressed 35S:*GFP* with 35S:*MdHIR1‐GFP* (left side of leaf) (Figures 6e and 7b,c). Moreover, trypan blue staining showed that *N. benthamiana* leaves that expressed 35S:*MdMRLK2‐GFP* with 35S:*MdHIR1‐GFP* showed less cell death than those that expressed 35S:*MdHIR1‐GFP* with 35S:*GFP* (Figure 6f). In addition, after inoculation with *Phytophthora capsici*, we found that areas of leaves that co‐expressed 35S:*MdMRLK2‐GFP* with 35S:*MdHIR1‐GFP* had larger lesions than those that co‐expressed 35S:*GFP* and 35S:*MdHIR1‐GFP* in *N. benthamiana* (Figure 6g). To test the function of *MdHIR1* during apple–*V. mali* interaction, we inoculated *V. mali* on apple leaves that co‐expressed 35S:*GFP* with 35S:*MdHIR1‐GFP* and 35S:*MdMRLK2‐GFP* with 35S:*MdHIR1‐GFP*. Areas of leaves that co‐expressed 35S:*MdMRLK2‐GFP* with 35S:*MdHIR1‐GFP* had larger lesions than those that co‐expressed 35S:*GFP* and 35S:*MdHIR1‐GFP* (Figure 7d). Taken together, these results suggest that *MdHIR1* positively regulates *P. capsici* and *V. mali* resistance. The interaction between MdMRLK2 and MdHIR1 limits MdHIR1‐mediated HR, and compromises *P*. *capsici* and *V. mali* resistance in leaves of *N. benthamiana* and apple, respectively.

### 2.6 MdMRLK2 impaired MdHIR1 self‐interaction

**FIGURE 8:**
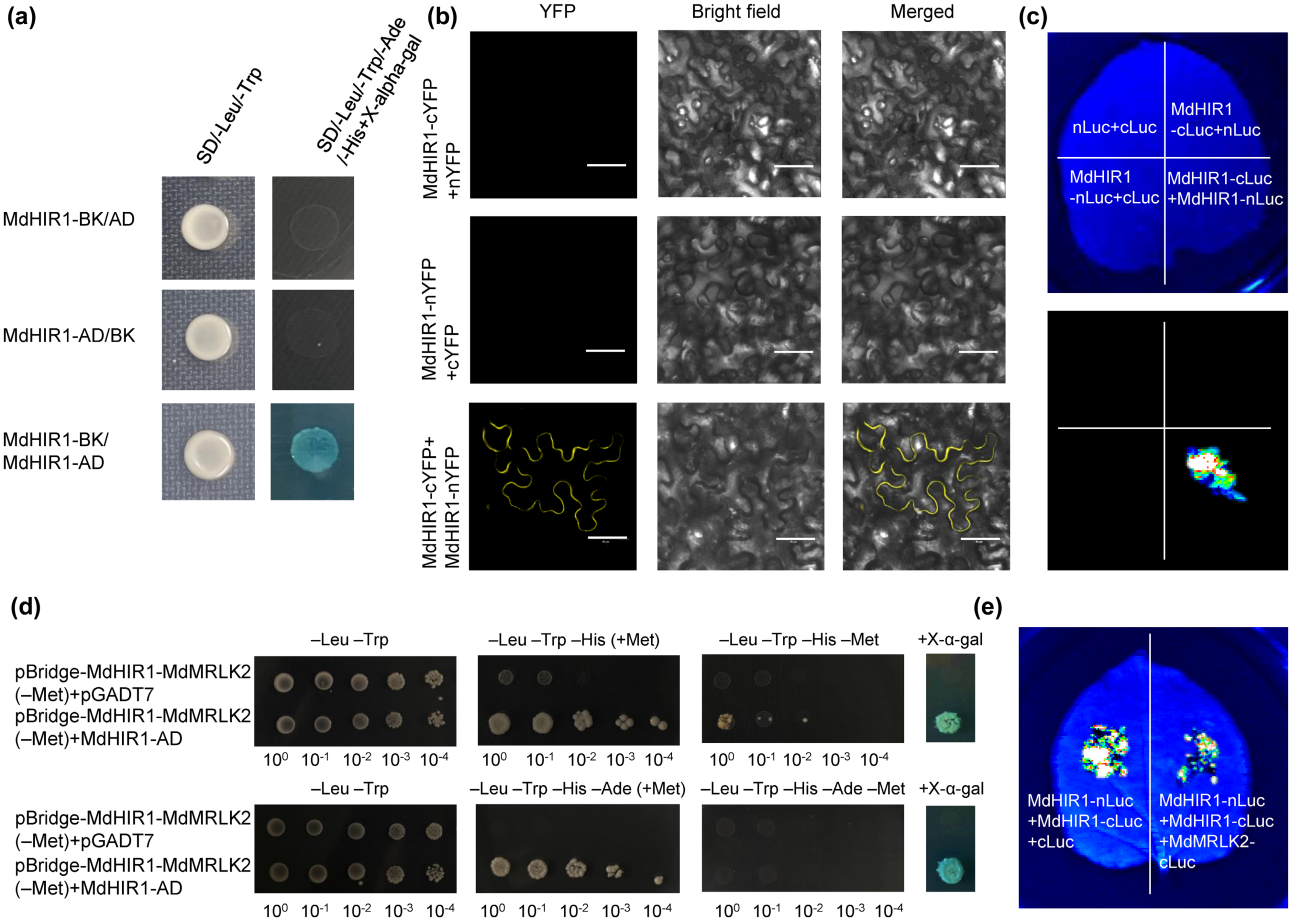
MdMRLK2 suppressed MdHIR1‐mediated hypersensitive reaction by impairing MdHIR1 self‐interaction. (a) A yeast two‐hybrid assay showed MdHIR1 self‐interaction. (b) Confirmation of MdHIR1 self‐interaction by bimolecular fluorescence complementation in *Nicotiana benthamiana* epidermal cells as indicated by a yellow fluorescence signal. nYFP, construct for YFP N‐terminal fusion expression; cYFP, construct for YFP C‐terminal fusion expression. Bar = 50 μm. (c) Confirmation of MdHIR1 self‐interaction by split‐luciferase complementation assay; the combinations of cLUC with nLUC, MdHIR1‐cLUC with nLUC, and MdHIR1‐nLUC with cLUC served as negative controls. (d) Yeast three‐hybrid growth assay to test the effect of MdMRLK2 on MdHIR1 self‐interaction. Yeast cells were grown in SD/−Leu/−Trp, SD/−Leu/−Trp/−His, and SD/−Leu/−Trp/−His/−Ade media with or without 1 mM methionine (Met) for 4 days. (e) The effect of MdMRLK2 on MdHIR1 self‐interaction in a split‐luciferase complementation assay

Y2H assays revealed that MdHIR1 was capable of self‐interaction (Figure 8a), and this self‐interaction was confirmed by BiFC and split‐luciferase complementation assays (Figure 8b,c). Because co‐expression of MdMRLK2 with MdHIR1 limited MdHIR1‐mediated HR, we wondered whether MdMRLK2 interfered with MdHIR1 self‐interaction. To test this possibility, we performed a yeast three‐hybrid (Y3H) assay by cloning *MdHIR1* and *MdMRLK2* into the pBridge plasmid to obtain pBridge‐MdHIR1‐MdMRLK2(−Met)‐BD, and we cloned MdHIR1 into pGADT7 to obtain MdHIR1‐AD. pBridge‐MdHIR1‐MdMRLK2(−Met)‐BD and MdHIR1‐AD were co‐expressed in the yeast strain Y2H Gold, and pBridge‐MdHIR1‐MdMRLK2(−Met)‐BD and pGADT7 were co‐expressed as a negative control. We then analysed yeast growth in SD/−Leu/−Trp/−His and SD/−Leu/−Trp/−His/−Ade media with or without 1 mM methionine (Met), which repressed the expression of *MdMRLK2*. Yeast cells expressing pBridge‐MdHIR1‐MdMRLK2(−Met)‐BD and MdHIR1‐AD grew well in SD/−Leu/−Trp/−His and SD/−Leu/−Trp/−His/−Ade media with Met, but they did not grow well in SD/−Leu/−Trp/−His medium and did not grow in SD/−Leu/−Trp/−His/−Ade medium without Met (Figure 8d). We also tested the effect of MdMRLK2 on MdHIR1 self‐interaction in a split‐luciferase complementation assay. The signal of leaves that co‐expressed MdMRLK2‐cLUC, MdHIR1‐nLUC, and MdHIR1‐cLUC was weaker than that of leaves that co‐expressed cLUC, MdHIR1‐nLUC, and MdHIR1‐cLUC (Figure 8e). All in all, these data demonstrate that MdMRLK2 has the capacity to disrupt MdHIR1 self‐interaction, thereby suppressing the HR mediated by MdHIR1.

## 3 DISCUSSION

Valsa canker, a destructive disease of apple trees, is caused by the ascomycete *V. mali* (Lee et al., 2006; Li et al., 2013; Wang et al., 2014). The pathogen typically invades apple trees through wounds or natural ostioles in the bark, and it induces severe tissue maceration and necrosis (Feng et al., 2021). Valsa canker was first identified in Japan and is now widespread in eastern Asia, where it causes severe yield losses each year and has a profound effect on apple production (Xu et al., 2020). However, the molecular mechanisms that underlie apple response to *V*. *mali* infection remain unclear.

FERONIA acts as a sensor of cell wall integrity during the host–pathogen interaction and triggers further downstream immune responses in the host cell (Ji et al., 2020). The immune responses triggered by FERONIA in response to fungal and bacterial pathogens were initially reported by Keinath and Kessler and colleagues (Keinath et al., 2010; Kessler et al., 2010). Previous studies have shown that two rice FERONIA‐like receptor genes, *OsFLR2* and *OsFLR11*, attenuate the resistance of rice seedlings to *Magnaporthe grisea* by down‐regulating defence‐related genes and suppressing ROS bursts (Yang et al., 2020). In *Arabidopsis*, FERONIA mutations confer increased resistance to *G*. *orontii* and *Fusarium oxysporum*, but reduce resistance to *Hyaloperonospora arabidopsidis* and *Colletotrichum higginsianum* (Kessler et al., 2010; Masachis et al., 2016). FERONIA has diverse functions in response to different pathogens based on their trophic type. In this study, *MRLK2* was highly induced by *V. mali* infection in twigs of a susceptible, but not of a resistant, *Malus* species, and *MdMRLK2* rapidly responded to *V. mali* in leaves and twigs of the *V. mali‐*susceptible cultivar Gala (Figure 1a–c). After inoculation with *V*. *mali*, 35S:*MdMRLK2* apple plants exhibited larger lesion areas than WT plants (Figure 1d–g), suggesting that MdMRLK2 negatively regulates apple tolerance to *V. mali*.

Hormones are key regulators of plant growth, development, and defences (Franck et al., 2018), and multiple hormone signalling pathways are integrated to boost plant immunity in response to biotic stresses. In *Arabidopsis*, FERONIA is involved in the crosstalk between several hormone pathways that regulate cell growth, seed yield, and stress responses (Franck et al., 2018; Liao et al., 2017). After plants were infected with *V. mali* in the present study, leaf ABA levels were 50.2% higher in the 35S:*MdMRLK2* lines than in the WT (Figure 2a), and twig ABA levels increased drastically in 35S:*MdMRLK2* lines compared with the WT (Figure 2b). By contrast, SA levels were lower in 35S:*MdMRLK2* than in the WT (Figure 2c,d). We also treated WT leaves with exogenous ABA and SA before *V. mali* inoculation, and the lesion areas were larger after ABA treatment and smaller after SA treatment compared with the control (Figure 2e), indicating that the increased ABA levels and reduced SA levels mediated by MdMRLK2 overexpression contributed to apple *V. mali* susceptibility. Similarly, the treatment of rice plants with ABA increased their susceptibility to black‐streaked dwarf mosaic virus by suppressing ROS accumulation and the JA pathway (Cui et al., 2017). Recent reports have demonstrated that the peptide ligand RALF23 acts through FERONIA to stabilize MYC2 and elevate JA signalling, negatively influencing plant immunity (Guo et al., 2018). In this study, there was no difference in JA content between 35S:*MdMRLK2* and WT plants (Figure S2).

Phenolic acids have been frequently described as contributing to defence against plant fungal pathogens, either through direct interference with the fungus or through reinforcement of plant structural components that act as a mechanical barrier (Gauthier et al., 2016; Lattanzio et al., 2006; Siranidou et al., 2002). In response to pathogen infection, phenolic acids are released from the cell wall or massively synthesized by the plant, accumulating rapidly at the infection sites (Atanasova‐Penichon et al., 2012). Among the phenolic acids, derivatives of cinnamic acid (e.g., caffeic, ferulic, and *p*‐coumaric acids) are the best recognized contributors to fusarium head blight resistance (Gauthier et al., 2015). Here, we measured phenolic acids in apple leaves and twigs, and found that the contents of phenolic acids (gallic acid, ferulic acid, *p*‐coumaric acid, and chlorogenic acid) were significantly higher in WT plants than in 35S:*MdMRLK2* plants (Figure 3). This result demonstrates that *MdMRLK2* overexpression is detrimental to polyphenol accumulation. Host protection against fungal pathogen invasion is due in large part to a defence system that is highly coordinated to prevent the spread of pathogens (Wang et al., 2013; Yu et al., 2016). PAL is a key enzyme in the phenylpropanoid pathway, which is responsible for aspects of the host defence system (Huang et al., 2010). CHT degrades chitin, which is the major component of fungal pathogen cell walls. GLU, one of the most fully characterized pathogenesis‐related proteins, also acts indirectly by releasing an oligosaccharide and eliciting defence reactions, then acting synergistically with CHT to inhibit fungal growth (Ji et al., 2021). Here, PAL, GLU, and CHT activities were all induced by *V*. *mali*, and this induction was greater in leaves and twigs of WT plants than in those of 35S:*MdMRLK2* plants after *V. mali* infection (Figure 4). This result indicates that MdMRLK2 plays a negative role in regulating defence‐related enzyme activities on *V. mali* infection.

To further clarify the mechanism by which MdMRLK2 overexpression promotes apple susceptibility to *V. mali*, we performed Y2H screening and found that MdMRLK2 targeted MdHIR1 (Figure 5a); BiFC and split‐luciferase complementation assays confirmed this interaction (Figure 5b,c). A co‐immunoprecipitation assay also indicated that MdMRLK2 interacted with MdHIR1 (Figure 5d). Induction of HIR genes occurs in response to attacks by various pathogens, including bacteria, fungi, and viruses, and the accumulation of HIR proteins induces host cell death and disease resistance (Duan et al., 2013; Jung & Hwang, 2007; Li et al., 2019b; Qi et al., 2011). The HR is defined as rapid cell death that occurs in the region of invasion; it limits pathogen spread, prepares the plant defence system for successive assaults, and is closely related to active resistance (Choi & Hwang, 2015; Noman et al., 2020; Pontier et al., 1998). Because MdMRLK2 interacts with MdHIR1, we hypothesized that this interaction might affect MdHIR1‐mediated HR and increase *V*. *mali* susceptibility in 35S:*MdMRLK2* plants. To test this possibility, we co‐expressed 35S:*GFP* with 35S:*MdHIR1‐GFP* and 35S:*MdMRLK2‐GFP* with 35S:*MdHIR1‐GFP* in *N*. *benthamiana* and apple leaves. Interestingly, 35S:*MdHIR1‐GFP* alone induced an HR and led to H_2_O_2_ accumulation and cell death (Figures 6c,e,f and 7a–c). However, 35S:*MdMRLK2‐GFP* co‐expressed with 35S:*MdHIR1‐GFP* limited MdHIR1‐mediated HR (Figures 6c and 7a) and suppressed *P. capsici* and *V. mali* resistance in *N*. *benthamiana* and apple leaves (Figures 6g and 7d). Previous studies have demonstrated that HIR1 exhibits self‐interaction (Jung & Hwang, 2007; Mei et al., 2020). Here, we confirmed the self‐interaction of MdHIR1 in apple by Y2H, BiFC, and split‐luciferase complementation assays (Figure 8a–c), and we performed Y3H and split‐luciferase complementation assays to demonstrate that MdMRLK2 impaired MdHIR1 self‐interaction (Figure 8d,e). Taken together, our data suggest that MdMRLK2‐mediated HR suppression is one possible mechanism by which *MdMRLK2* overexpression reduces *V*. *mali* resistance.

**FIGURE 9:**
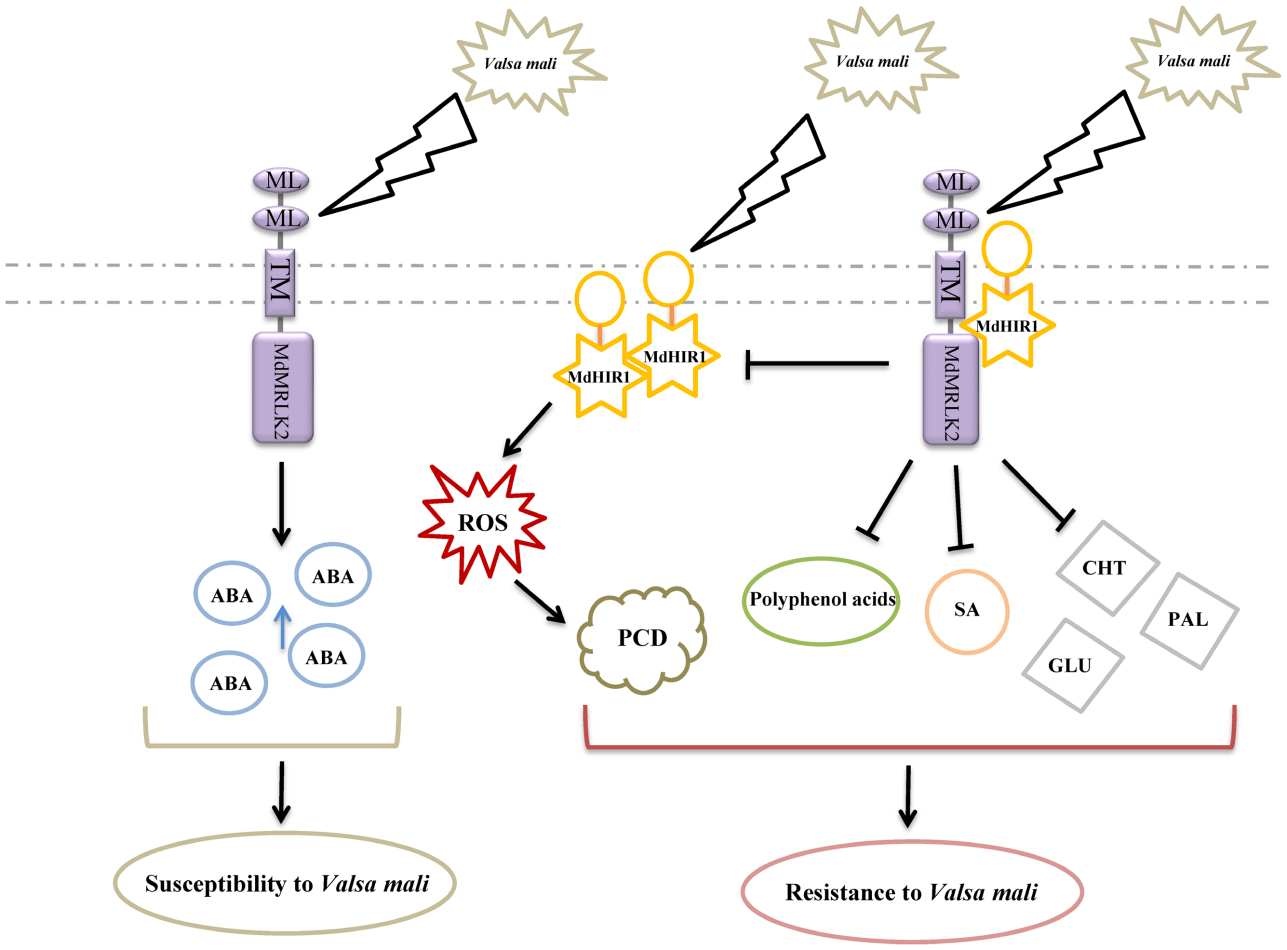
Proposed model of the regulatory mechanism by which *MdMRLK2* overexpression alters the response to *Valsa mali* in apple

We found that *MdMRLK2* overexpression compromised Valsa canker resistance in apple by suppressing HR and altering hormone and polyphenol levels (Figure 9). Hormone and polyphenol levels play an important role in plant resistance. After *V*. *mali* inoculation, 35S:*MdMRLK2* apple plants had higher ABA and lower SA levels, reduced polyphenol accumulation, and lower PAL, GLU, and CHT activities compared with the WT. Interestingly, a direct interaction existed between MdMRLK2 and MdHIR1. Moreover, MdMRLK2 impaired MdHIR1 self‐interaction and limited MdHIR1‐mediated HR. Overall, our findings reveal a novel function of the apple FERONIA receptor‐like kinase MdMRLK2 in defence against Valsa canker disease.

## 4 EXPERIMENTAL PROCEDURES

### 4.1 Materials and treatments

GL‐3, isolated from cv. Royal Gala, was used for apple transformation. The transformation method was similar to that described by Dai et al. (2013). Tissue‐cultured WT and transgenic apple plants were subcultured every 4 weeks. The rooting method for WT and transgenic plants was based on that described in Sun et al. (2018). The rooted WT and transgenic apple plantlets were cultivated on rooting medium for 40 days and then transferred to pots (8 × 8 cm) that contained nutrient soil, vermiculite, and perlite mixed in a 3:1:1 ratio. After 30 days of cultivation, plants were moved into larger plastic pots (30 × 18 cm) filled with forest soil, sand, and organic fertilizer (5:1:1 by volume) and maintained in a glasshouse. After acclimation and growth with half‐strength Hoagland's nutrient solution irrigation, 1‐year‐old twigs and uniformly sized healthy leaves from WT, 35S:*MdMRLK2*, *M. yunnaensis*, and *M. mellana* apple trees were collected from the Horticultural Experimental Station of Northwest A&F University, Yangling (34°20′N, 108°24′E), China, in August 2020.

*V. mali* isolate 03‐8 and *P*. *capsici* were provided by the Laboratory of Integrated Management of Plant Diseases, College of Plant Protection of Northwest A&F University. For 03‐8 activation, the isolate was cultivated on potato dextrose agar at 25°C for 3 days. *P*. *capsici* was cultivated on carrot dextrose agar at 25°C for 3 days.

Inoculation was performed as described by Suzaki et al. (1997) with the minor modifications mentioned by Feng et al. (2020). Prior to inoculation, fully expanded apple leaves were surface‐disinfected with 0.6% sodium hypochlorite solution and rinsed three times by spraying with sterile water. *V. mali* strain 03‐8 was cultured on potato dextrose agar for 3 days. Agar plugs (5 mm each) were taken from the margin of the growing colony and placed on the abaxial leaf surface by the needle‐stab method. Twigs were cut into 20‐cm segments and washed with tap water, immersed in 0.6% sodium hypochlorite for 6 min, and rinsed with sterile water three times. The ends of the twigs were sealed with wax. Each twig segment was subjected to wounding with a hole puncher (Xu et al., 2018). Agar plugs without fungus were used as negative controls. The twigs and leaves were placed horizontally in a plastic box, which was immediately covered with a vinyl film to retain humidity at 25°C. Inoculated leaves were incubated for 3 days and inoculated twigs were incubated for 5 days. Leaf lesion sizes were measured by the crossing method, and lesion areas were calculated based on the diameter. The total lengths of longitudinal lesions along twigs were measured directly to determine the size of the lesions. The leaves and bark were immediately collected, frozen in liquid nitrogen, and stored at −80°C.

GL‐3 leaves were sprayed with 100 μM ABA or 300 μM SA for 6 h, then inoculated with *V. mali* on the abaxial surface by the needle‐stab method. Leaves sprayed with sterile water served as controls.

For transient gene expression, leaves from 2‐month‐old GL‐3 plants were used for agroinfiltration. For *V. mali* infection on apple calli, agar plugs (5 mm each) taken from the margin of the growing colony were placed on 20‐day‐old strongly growing calli of the WT and three *MdMRLK2* RNAi lines.

*N. benthamiana* plants were grown and maintained in plant growth chambers at an ambient temperature of 23°C under a 16‐h light/8‐h dark photoperiod. Leaves of 5‐week‐old *N. benthamiana* were used for agroinfiltration and *P*. *capsici* infection. For mycelial inoculation, 5‐mm disks of 3‐days' growth medium were inoculated onto *N. benthamiana* leaves. The inoculated leaves were photographed at 36 or 48 h postinoculation.

### 4.2 Transient expression in *N. benthamiana*

*Agrobacterium tumefaciens* GV3101 was used to express MdMRLK2‐FLAG, MdMRLK2‐GFP, and MdHIR1‐GFP fusion proteins by agroinfiltration as described by Huo et al. (2021).

### 4.3 RNA extraction and reverse transcription‐quantitative PCR

Total RNA was extracted using the Wolact Plant RNA Isolation Kit (Wolact) according to the manufacturer's instructions. cDNA synthesis was performed using the RevertAid First Strand cDNA Synthesis Kit (Thermo Scientific). Quantitative PCR was performed using SYBR premix ExTaq (Takara) on a CFX96 Touch Real‐Time PCR Detection System (Bio‐Rad). Fold changes in gene expression were calculated using the 2^−ΔΔ^ *^C^* ^t^ method, and *MdMDH* was used as the endogenous control. All primers are listed in Table S1.

### 4.4 Measurement of hormones, polyphenols, and disease resistance enzyme activities

ABA and SA contents were measured using the method of Zhou et al. (2019). In brief, 100 mg samples were ground in 2 ml of extraction solution (20:79:1 methanol:isopropanol:acetic acid), then shaken for 5 min and incubated at 4°C for 12 h. The samples were centrifuged at 13,500 × *g* for 10 min at 4°C and filtered through a 0.22‐μm organic filter prior to high performance liquid chomatography‐mass spectrometry (HPLC‐MS) analysis. For polyphenol measurement, 100 mg samples were ground in 1 ml of extraction solution (25:24:1 methanol:water:formic acid) with ultrasonication (25°C, 40 Hz, 100 W) for 20 min, then shaken (25°C, 150 rpm) for 20 min. The samples were centrifuged at 10,000 × *g* for 15 min, and the supernatant was analysed by HPLC‐MS after filtering through a 0.22‐μm organic filter and diluting fivefold. GLU, CHT, and PAL activities were measured using colourimetric assay kits (Suzhou Comin Biotechnology Co., Ltd).

### 4.5 Detection of H_2_O_2_ and cell death in *N. benthamiana* leaves

The production of H_2_O_2_ and ∙O_2_^−^ in leaves was detected visually using 3,3′‐diaminobenzidine‐HCl (DAB) and nitrotetrazolium blue chloride (NBT) as substrates. For H_2_O_2_ staining, leaves were incubated for 8 h in the dark at 25°C in the presence of 1 mg/ml DAB (pH 3.8). For ∙O_2_^−^ staining, leaves were incubated for 1 h at 25°C in the presence of 1 mg/ml NBT (pH 7.5). Leaves were then immersed in boiling 80% ethanol for 20 min, cooled, and preserved in 10% glycerol at 25°C.

Cell death was detected in *N. benthamiana* leaves by trypan blue staining. Excised leaves were boiled for 5 min in a 0.021 mM trypan blue solution (containing 0.137 M glycerol, 0.131 M lactic acid, 0.106 M phenol), diluted threefold with ethanol, and further incubated for 1 day. The *N. benthamiana* leaves were destained in a 2.5 g/ml chloral hydrate solution for 6 h, the solution was replaced for further destaining for 24 h, and the samples were preserved in 10% glycerol at room temperature and photographed.

### 4.6 Y2H and Y3H assays

A partial coding sequence for the MdMRLK2 protein (469–892 amino acids) was cloned into the pGBKT7 vector as bait for Y2H screening. Y2HGold yeast cells were first transformed with pGBKT7‐*MdMRLK2*. The coding sequence of *MdHIR1* was inserted into the pGADT7 and pGBKT7 vectors (primers are listed in Table S1). The Y2H assay was performed as described by Petzold et al. (2018). The transformed yeast clones were first grown on SD/−Leu/−Trp medium and then transferred onto SD/−Leu/−Trp/−His/−Ade medium. Combinations of pGBKT7‐*MdMRLK2*, pGBKT7‐*MdGHIR1*, and pGADT7‐*MdHIR1* were used to detect their potential interactions, and the combination of pGBKT7‐*MdMRLK2*, *pGBKT7*‐*MdHIR1*, and *pGADT7* was used as a negative control.

For the Y3H assay, the coding sequence of *MdHIR1* was inserted into the multiple cloning site (MCS) Ⅰ of the pBridge plasmid using the *Eco*RI/*Bam*HI sites, and *MdMRLK2* was inserted into the MCS Ⅱ of the pBridge plasmid using the *Not*I/*Bgl*II sites. For the growth assay, yeast cells containing pBridge*‐MdHIR1‐MdMRLK2* and pGADT7‐*MdHIR1* or pBridge‐*MdHIR1‐MdMRLK2* and pGADT7 were co‐transformed into Y2HGold, and the transformed cells were selected on synthetic dropout selection medium lacking Leu and Trp. Three days after transformation, the cells were transferred to medium lacking Leu, Trp, and His, with or without Met, and/or lacking Leu, Trp, His, and Ade, with or without Met, for 4 days. pBridge*‐MdHIR1‐MdMRLK2* and pGADT7 were used as the control.

### 4.7 BiFC assay

The coding sequences of *MdMRLK2* and *MdHIR1* were inserted into the 35S:pSPYCE‐*cYFP* vector, and the coding sequence of *MdHIR1* was inserted into the 35S:pSPYNE*‐nYFP* vector. We used an apple aquaporin protein MdPIP2, a plasma membrane protein, as negative control. Leaves from 5‐week‐old *N. benthamiana* were used for the BiFC assay, and fluorescence was detected as described by Wang et al. (2020). Confocal imaging was performed using an FV3000 confocal laser scanning microscope (Olympus). The primers used for vector construction are listed in Table S1.

### 4.8 Split‐luciferase complementation assay

The coding sequence of *MdHIR1* without the stop codon was inserted into pCAMBIA1300‐nLUC, and the coding sequences of *MdMRLK2* and *MdHIR1* were cloned into pCAMBIA1300‐cLUC. The split‐luciferase complementation assay was performed by transient expression in leaves of *N. benthamiana* by agroinfiltration as described by Fernandez et al. (2020). Leaves that co‐expressed different constructs were examined for luciferase activity by applying 1 mM d‐luciferin and placing them in the dark for 5 min before imaging. Luciferase complementation was observed with a CCD imaging system (Lumazone Pylon 2048B) using 10‐min exposures.

### 4.9 Co‐immunoprecipitation assay

The coding sequence of *MdMRLK2* was inserted into pCambia‐4×Myc‐MCS‐3×FLAG to generate the MdMRLK2‐FLAG construct. The coding region of *MdHIR1* was inserted into pGWB405‐GFP to obtain MdHIR1‐GFP. MdMRLK2‐FLAG and pCAMBIA35S‐4×Myc‐MCS‐3×FLAG were co‐expressed with MdHIR1‐GFP in *N. benthamiana* leaves. Total proteins were extracted in extraction buffer (20 mM Tris pH 7.5, 150 mM NaCl, 1 mM EDTA, 1 mM EGTA, 1% Triton X‐100, 2.5 mM sodium pyrophosphate, 1 mM β‐glycerophosphate, 1 mM Na_3_VO_4_, 1 μg/ml leupeptin, and 1 mM phenylmethylsulfonyl fluoride). Anti‐FLAG M2 antibody conjugated with Sepharose beads was incubated with the extracted proteins at 4°C with gentle shaking overnight. The precipitated samples were washed four times with protein extraction buffer and eluted by adding 3× SDS protein loading buffer and boiling for 5 min to obtain proteins for western blotting. Each immunoblot was incubated with the appropriate primary antibody (anti‐FLAG antibody 1:2000, anti‐GFP antibody 1:2000) overnight at 4°C. Immunoblots were developed using horseradish peroxidase‐conjugated mouse secondary antibody at a 1:2000 dilution (Proteintech Group, Inc.) and imaged with a chemiluminescence detection system.

### 4.10 Statistical analysis

The data were analysed using one‐way analysis of variance followed by Tukey's multiple comparison test (*p* < 0.05) in SPSS 20.0 (IBM).

## CONFLICT OF INTEREST

The authors declare that they have no conflict of interest.

## Supporting information


**FIGURE S1** Knocking down *MdMRLK2* improved the resistance to *Valsa mali* in apple calli. (a) PCR confirmation of transgenic apple calli. Lanes: M, molecular marker DL2000; WT, nontransformed wild type; RNAi‐5, RNAi‐6, and RNAi‐7, *MdMRLK2* RNAi transgenic apple calli. (b) Reverse transcription‐quantitative PCR analysis of *MdMRLK2* transcripts in lines RNAi‐5, RNAi‐6, and RNAi‐7. (c) The phenotypes of WT and *MdMRLK2* RNAi apple calli lines inoculated with *V. mali* at 3 days postinoculation (dpi). Fifteen plates with apple calli were used as biological replicates. (d) Cell death staining with trypan blue in WT and *MdMRLK2* RNAi apple calli lines after inoculation with *V. mali* at 3 dpi. Fifteen plates with apple calli were used as biological replicates

**FIGURE S2** Relative mRNA levels of *MdPR1*, *MdPR4*, *MdPR5*, and *MdPAL* in (a) leaves and (b) twigs of wild‐type (WT) and 35S:*MdMRLK2* transgenic plants (OE‐1 and OE‐2)

**FIGURE S3** Jasmonic acid (JA) contents in (a) leaves and (b) twigs of wild‐type (WT) and 35S:*MdMRLK2* transgenic plants (OE‐1 and OE‐2)

**FIGURE S4** No interaction was observed between MdMRLK2 and a plasma membrane‐localized aquaporin MdPIP2 using bimolecular fluorescence complementation assay in *Nicotiana benthamiana* epidermal cells. nYFP, the construct for YFP N‐terminal fusion expression; cYFP, the construct for YFP C‐terminal fusion expression. Bar = 50 μm

**FIGURE S5** Subcellular localization of MdHIR1. AtCBL1n served as a plasma membrane marker. Bar = 50 μm

**TABLE S1** Application of primers and sequences

## Data Availability

The data supporting the findings of this study are available from the corresponding author upon reasonable request.
