# Supplementary figures and images for: The apple FERONIA receptor‐like kinase MdMRLK2 negatively regulates Valsa canker resistance by suppressing defence responses and hypersensitive reaction
Source: Mol Plant Pathol. 2022 Apr 12;23(8):1170–86. doi: 10.1111/mpp.13218 (PMC9276949; doi:10.1111/mpp.13218)

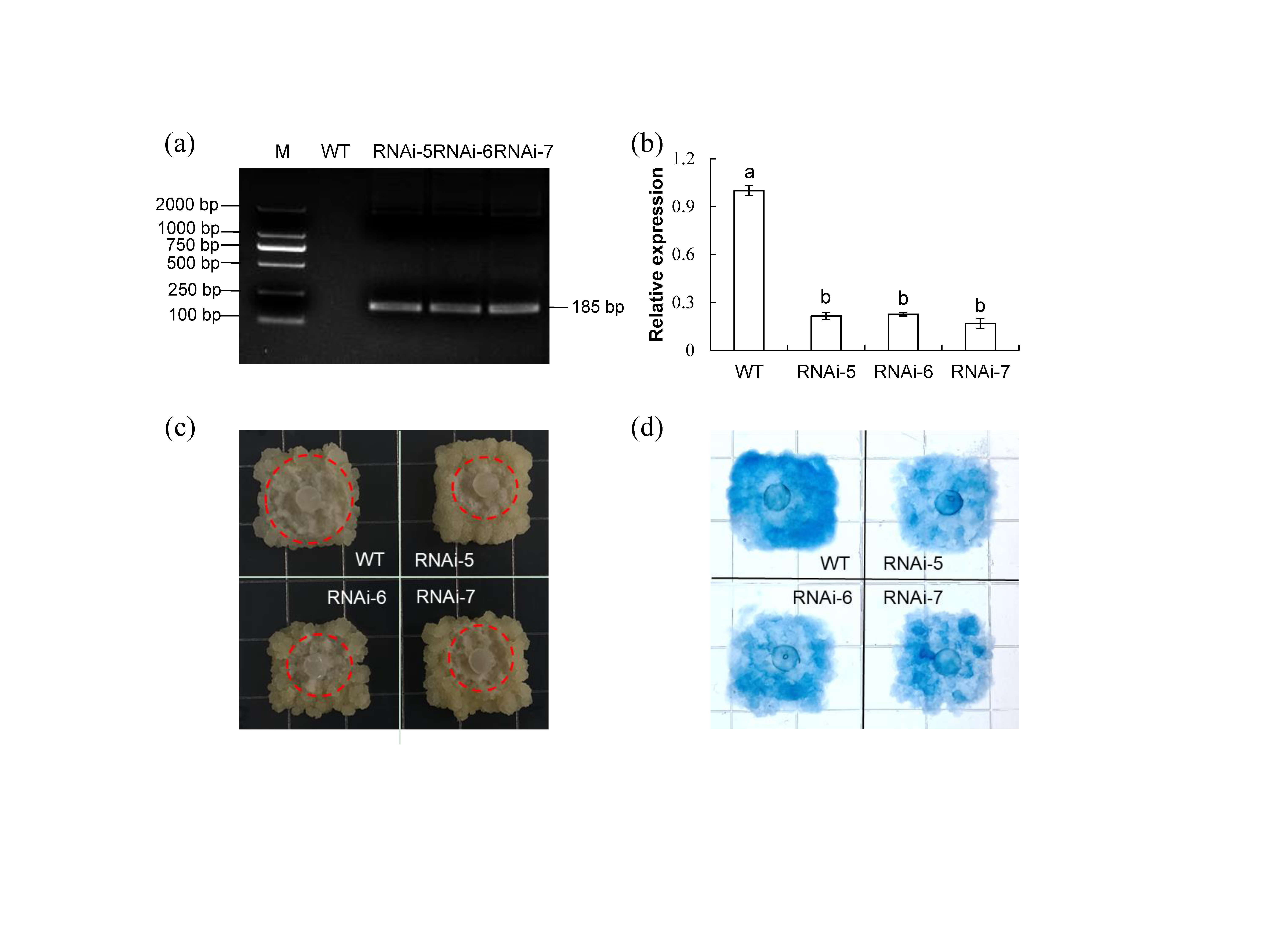

Supplement: Supplementary file 1 — FIGURE S1 Knocking down MdMRLK2 improved the resistance to Valsa mali in apple calli. (a) PCR confirmation of transgenic apple calli. Lanes: M, molecular marker DL2000; WT, nontransformed wild type; RNAi‐5, RNAi‐6, and RNAi‐7, MdMRLK2 RNAi transgenic apple calli. (b) Reverse transcription‐quantitative PCR analysis of MdMRLK2 transcripts in lines RNAi‐5, RNAi‐6, and RNAi‐7. (c) The phenotypes of WT and MdMRLK2 RNAi apple calli lines inoculated with V. mali at 3 days postinoculation (dpi). Fifteen plates with apple calli were used as biological replicates. (d) Cell death staining with trypan blue in WT and MdMRLK2 RNAi apple calli lines after inoculation with V. mali at 3 dpi. Fifteen plates with apple calli were used as biological replicates [file MPP-23-1170-s006.tiff]

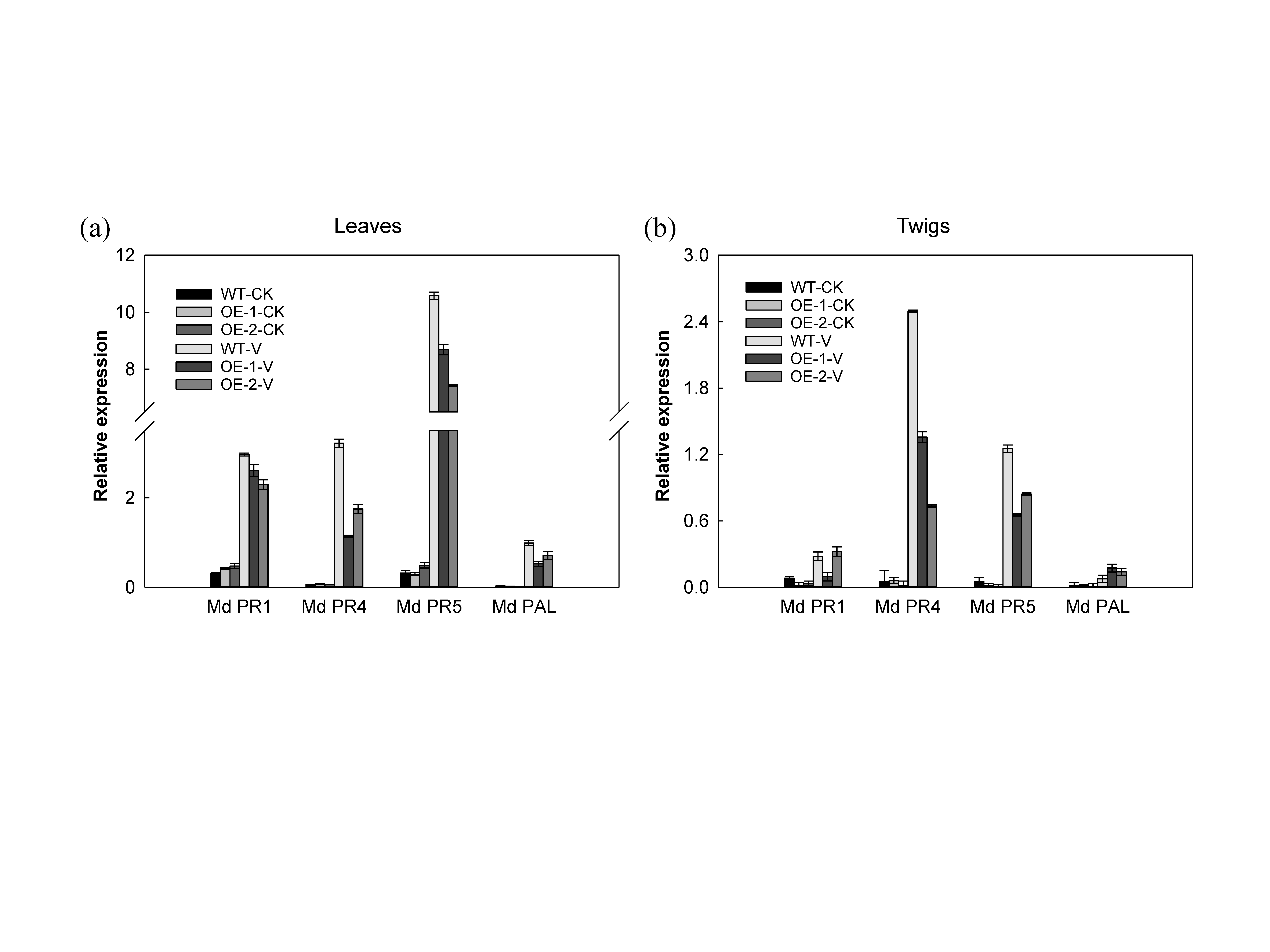

Supplement: Supplementary file 2 — FIGURE S2 Relative mRNA levels of MdPR1, MdPR4, MdPR5, and MdPAL in (a) leaves and (b) twigs of wild‐type (WT) and 35S:MdMRLK2 transgenic plants (OE‐1 and OE‐2) [file MPP-23-1170-s001.tiff]

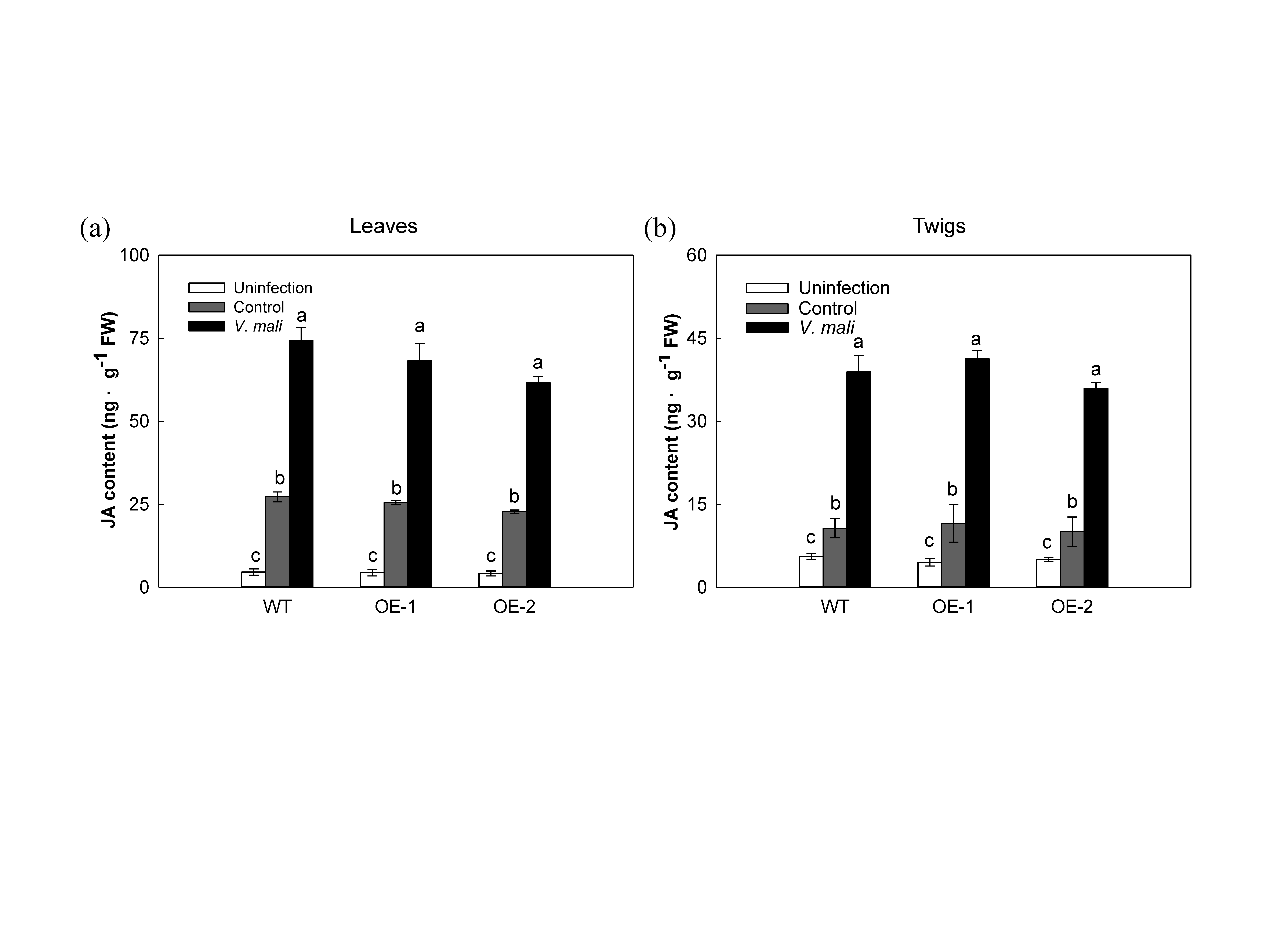

Supplement: Supplementary file 3 — FIGURE S3 Jasmonic acid (JA) contents in (a) leaves and (b) twigs of wild‐type (WT) and 35S:MdMRLK2 transgenic plants (OE‐1 and OE‐2) [file MPP-23-1170-s003.tiff]

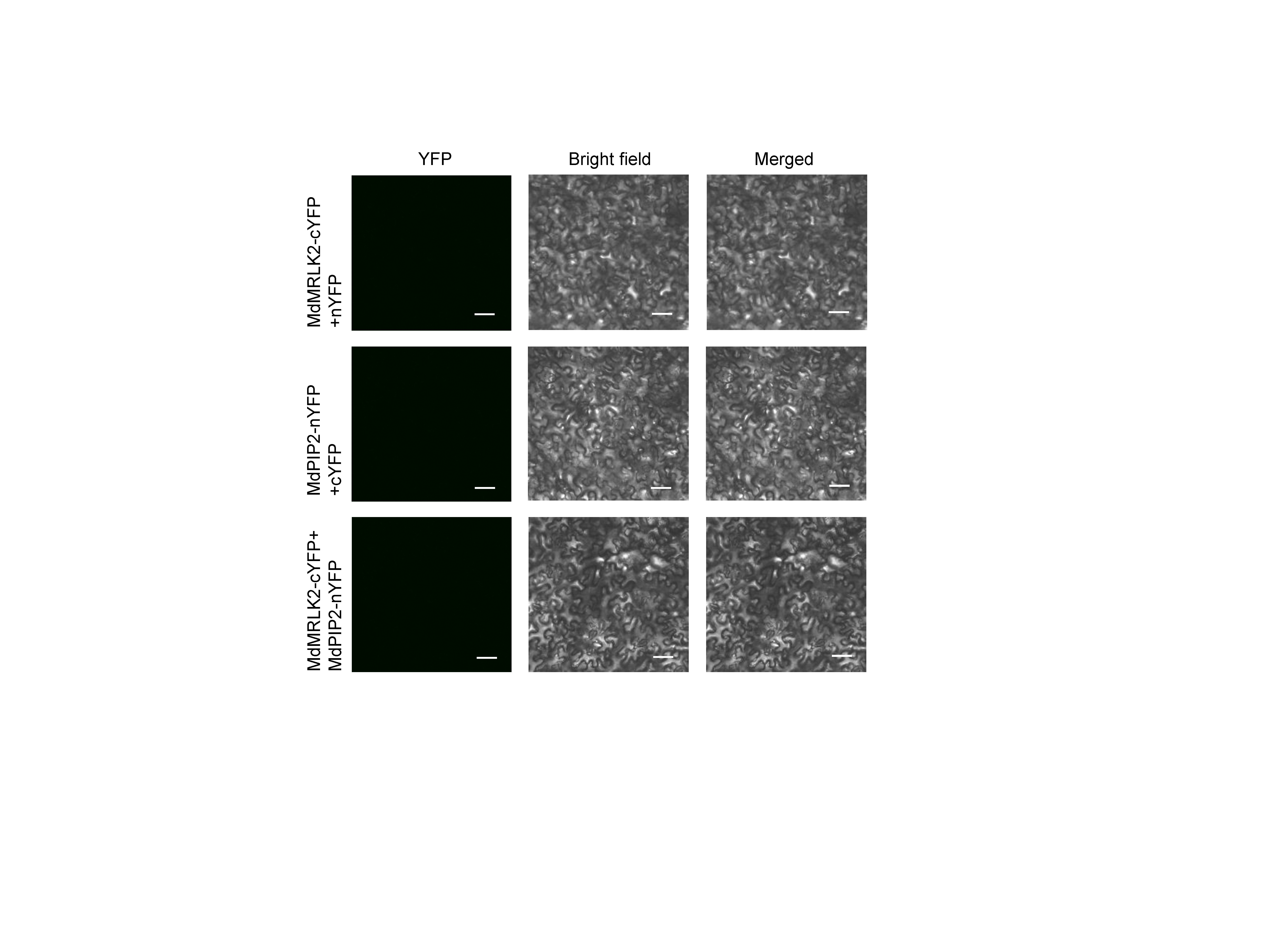

Supplement: Supplementary file 4 — FIGURE S4 No interaction was observed between MdMRLK2 and a plasma membrane‐localized aquaporin MdPIP2 using bimolecular fluorescence complementation assay in Nicotiana benthamiana epidermal cells. nYFP, the construct for YFP N‐terminal fusion expression; cYFP, the construct for YFP C‐terminal fusion expression. Bar = 50 μm [file MPP-23-1170-s002.tiff]

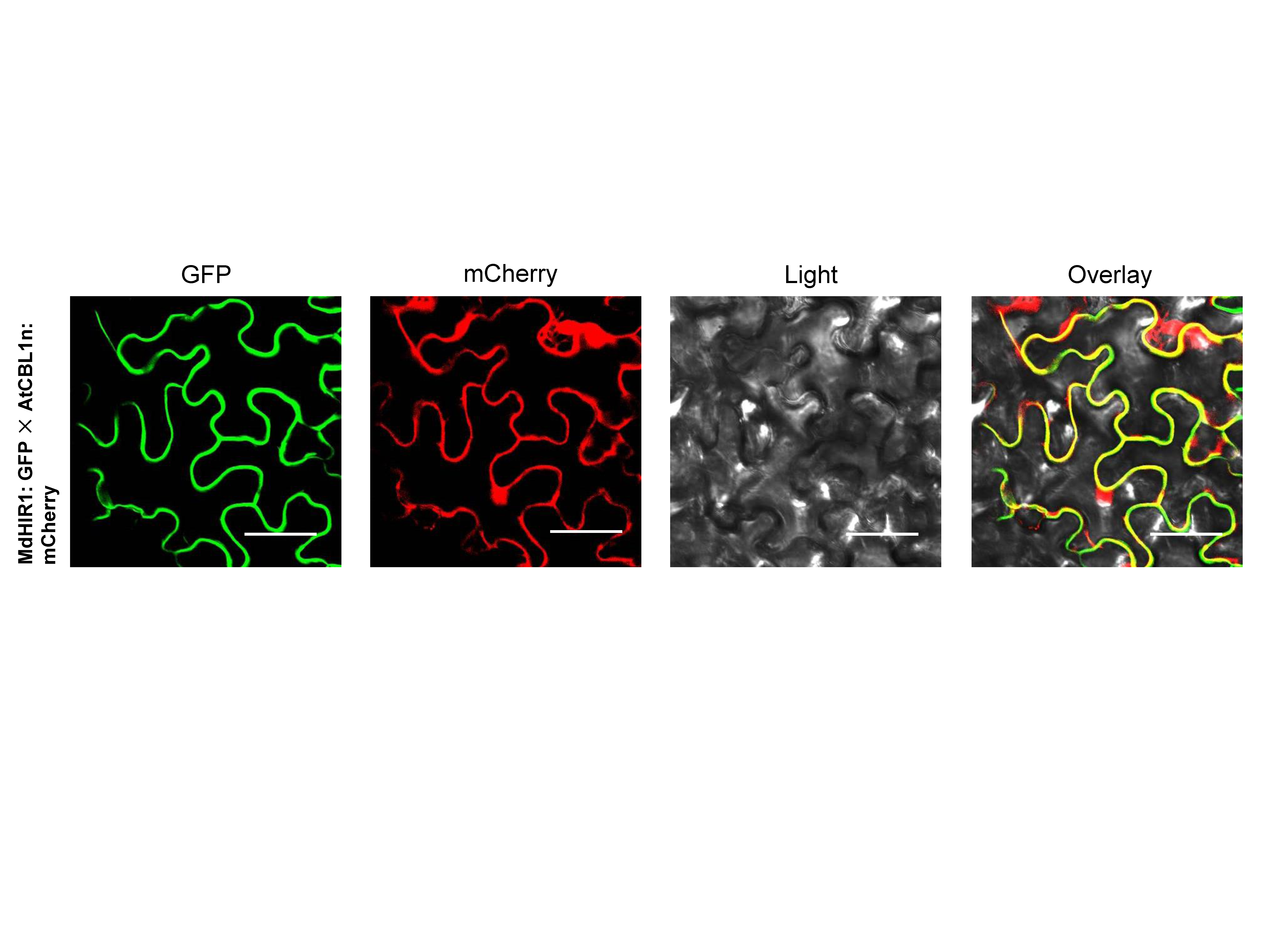

Supplement: Supplementary file 5 — FIGURE S5 Subcellular localization of MdHIR1. AtCBL1n served as a plasma membrane marker. Bar = 50 μm [file MPP-23-1170-s005.tiff]
