# Supplementary material for: The apple FERONIA receptor‐like kinase MdMRLK2 negatively regulates Valsa canker resistance by suppressing defence responses and hypersensitive reaction
Source: Mol Plant Pathol. 2022 Apr 12;23(8):1170–86. doi: 10.1111/mpp.13218 (PMC9276949; doi:10.1111/mpp.13218)
Supplement: Supplementary file 6 — TABLE S1 Application of primers and sequences [file MPP-23-1170-s004.docx]

Table S1. Application of primers and sequences

| **Primer Name** | **Purpose/Vector** | **Sequence (5’-3’)** |
| --- | --- | --- |
| *MdMRLK2* | **Clone/pMD19T-simple** | F: ATGAACACTGCTGGGTTTGTAGCTG  R: CTAACGTCCTTTAGGCTGCATGATT |
| *MdHIR1* | **Clone/pMD19T-simple** | F: ATGGGTAATCTATTCTGTTGTGTTCA  R: CTACTGATAAGAACCCTGAAGAAGTCC |
| *MdMRLK2*  *MdMRLK2*  *MdHIR1*  *MdMRLK2*  *MdHIR1*  *MdMRLK2*  *MdHIR1*  *MdPAL*  *MdPR1*  *MdPR4*  *MdPR5*  *MDH* | **PGWB405**  **PSPYCE/YNE**  **PSPYCE/YNE**  **PGBKT7**  **PGADT7**  **qRT-PCR** | F: CGAGCTCGGTACCCGGGGATCCATGAACACTGCTGGGTTTGTAGCTG  R: CCTTGCTCACCATGGTGTCGACACGTCCTTTAGGCTGCATGATT  F: GCCTGGCGCGCCACTAGTGGATCCATGGCATCCGCCGAGAATACT  R: GGGAGCGGTACCCTCGAGGTCGACTCCTTTAGGCTGCATGAT  F: GCCTGGCGCGCCACTAGTGGATCC ATGGGTAATCTATTCTGTTGTGTTCA  R: GGGAGCGGTACCCTCGAGGTCGACCTGATAAGAACCCTGAAGAAGTCC  F: GGCCATGGAGGCCGAATTCTCTCGCCGTCGTAGACAAC  R: GCTGCAGGTCGACGGATCCCTAACGTCCTTTAGGCTG  F: CATGGAGGCCAGTGAATTC ATGGGTAATCTATTCTGTTGTGTTCA  R: GCTCGAGCTCGATGGATCC CTACTGATAAGAACCCTGAAGAAGTCC  F: AGTTAGTGGTGGAATTGCCGTAGC  R: TGAACTTGCGTCCTTCCGTTGTC  F: GTGGCAACTAATGAGAAGGCAGAGG  R: ACCCAGCCCAGAGAGATACTTTGAC  F: GACCAAACGGTCAGACCCTCAATG  R: AGCCAAGCCAGAACCAACAGCAG  F: GCAGCAGTAGGCGTTGGTCCCT  R: CCAGTGCTCATGGCAAGGTTTT  F: CCACCTCTACAATCCACAGCAAAAC  R: GTCCGCAAAAGGCAGTCCATCCA  F: AACTAGCATCCAAAGCTAGCC  R: CCACAGTCTGCAGTTTCACAAG  F: ATTCAAGTATGCCTGGGTGC  R: CAGTCAGCCTGTGATGTTCC |
